# Antibody–Drug Conjugates and Peptide–Drug Conjugates: Current Understandings and Future Perspectives

**DOI:** 10.1002/mco2.70897

**Published:** 2026-08-05

**Authors:** Ruxi Zheng, Shuiquan Li, Zhigang Zhang, Mengke Niu, Jing Fei, Jiaojiao Zhang, Jianwei Zhou, Kongming Wu, Ming Yi, Tianye Li

**Affiliations:** ^1^ Department of Gynecology The Second Affiliated Hospital Zhejiang University School of Medicine Zhejiang University Hangzhou China; ^2^ Zhejiang Provincial Clinical Research Center For Gynecological Diseases Hangzhou China; ^3^ Department of Rehabilitation and Traditional Chinese Medicine The Second Affiliated Hospital Zhejiang University School of Medicine Zhejiang University Hangzhou China; ^4^ Department of Breast Surgery The First Affiliated Hospital College of Medicine Zhejiang University Hangzhou China; ^5^ Key Laboratory of Cancer Invasion and Metastasis Ministry of Education Tongji Hospital Tongji Medical College Huazhong University of Science and Technology Wuhan China; ^6^ Cancer Center Shanxi Bethune Hospital Shanxi Academy of Medical Science Tongji Shanxi Hospital Third Hospital of Shanxi Medical University Taiyuan China

**Keywords:** antibody–drug conjugate, cancer, peptide–drug conjugate

## Abstract

Antibody–drug conjugates (ADCs) and peptide–drug conjugates (PDCs) are modular targeted therapeutics in which molecular recognition is coupled with controlled payload delivery. Owing to their high specificity and precision, they have been regarded as a transformative paradigm for cancer therapy. In this review, current understandings and future perspectives of ADCs and PDCs are synthesized across rational design principles, historical evolution, clinical translation, and emerging formats, aiming to improve practical comprehension and application in oncology. Key determinants of performance: target selection, antibody or peptide carrier attributes, linker stability and cleavage mechanisms, payload classes, and conjugation strategies, are additionally discussed. Milestone advances achieved spanning hematologic malignancies and solid tumors are emphatically summarized to provide a framework by which future conjugate development may be contextualized. In parallel, the expansion of PDCs as complementary platforms is outlined, and advantages are highlighted, while limitations are also considered. ADCs and PDCs still face challenges such as toxicity, resistance, and poor stability. However, through breakthroughs including linker optimization, novel payloads, bispecific designs, and AI‐driven approaches, future conjugated drugs will continue to evolve toward personalization, combination therapies, and broader indications. This article provides a systematic synthesis of extant research, thereby facilitating the future advancement of targeted conjugate drugs toward greater precision and individualization. It holds significant academic value as a reference resource and offers practical guidance for the development of novel antineoplastic therapeutics.

## Graphical abstract

The graphic summarizes the design principles, intracellular trafficking, clinical progress, challenges, and future directions of antibody–drug conjugates (ADCs) and peptide–drug conjugates (PDCs). ADCs consist of a tumor‐targeting antibody, a chemical linker, and a potent payload, whereas PDCs use peptide ligands to deliver cytotoxic, radionuclide, or other therapeutic/diagnostic cargos. After target recognition and binding, these conjugates are internalized through endocytosis, processed in the endolysosomal compartment, and release their payloads to induce tumor‐cell killing. Membrane‐permeable payloads may also diffuse into adjacent tumor cells and mediate a bystander effect. Representative ADCs and PDCs have achieved clinical success in hematologic malignancies and solid tumors, with continued advances improving efficacy, safety, and indication coverage. Key limitations include off‐target toxicity, drug resistance, heterogeneous target expression, linker instability, manufacturing complexity, and limited tumor penetration, particularly for large ADCs. Future development is expected to focus on optimized linkers and conjugation strategies, next‐generation payloads, bispecific and multifunctional conjugates, rational combination therapies, patient selection, broader indications, and AI/ML‐assisted molecular design.

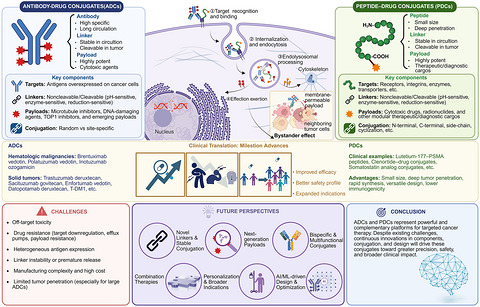

## 1 Introduction

Cancer has always been a focal point of global research due to its well‐documented economic and social burdens. In 2022, approximately 20 million new cancer cases and 9.7 million cancer‐related deaths were reported worldwide. Projections indicate that the number of new cancer cases may rise to 35 million by 2050 [1]. These statistics continue to drive advancements in antitumor therapies.

Traditional antitumor therapies include surgery, radiotherapy, and chemotherapy. Surgery represents the most historically established and fundamental local treatment approach, targeting the direct excision of solid tumors [2]. For early‐stage, localized solid tumors without metastasis, surgery may achieve curative outcomes. However, surgery constitutes an invasive procedure carrying inherent risks [3], and its value is limited for hematological malignancies or advanced tumors with extensive metastasis. Moreover, the risk of metastasis could be increased by the surgical procedure [4]. Radiotherapy employs ionizing radiation to locally irradiate tumors, disrupting tumor cell DNA to eliminate the neoplasm. Nevertheless, radiotherapy carries localized side effects, with normal tissues also susceptible to damage [5]. Chemotherapy remains indispensable for adjuvant therapy, disseminated cancers and many hematologic malignancies, yet its lack of tumor selectivity frequently causes systemic toxicities, including myelosuppression and gastrointestinal injury [6, 7].

With advances in molecular oncology and immunology, cancer care has transitioned toward precision medicine driven by targeted therapies and immunotherapy [8, 9, 10]. Targeted therapy employs specific drugs to deliver a “precision strike” against cancer cells by targeting their unique genetic mutations or molecular markers. In this context, drug conjugates have emerged as a compelling strategy that integrates molecular recognition with potent therapeutic payloads [11, 12, 13]. Antibody–drug conjugates (ADCs) exemplify this paradigm by combining the tumor‐homing specificity of monoclonal antibodies (mAbs) with the cytotoxic strength of highly potent small‐molecule drugs, linked through engineered chemistries designed to control stability in circulation and payload release in tumors [14]. By spatially restricting drug delivery, ADCs aim to widen the therapeutic window relative to conventional chemotherapy and have reshaped treatment landscapes across hematological malignancies and multiple solid tumors [15]. This approach has pioneered a new paradigm in tumor treatment. The intellectual roots of ADCs trace back to Paul Ehrlich's “magic bullet” concept, but clinical translation required concurrent breakthroughs in antibody engineering, linker design, and payload discovery [16]. Since the approval of the first ADC drug, Mylotarg [17], in 2000, the field has progressed from the setbacks of first‐generation products to the success of second‐generation ones, culminating in the explosive development of third‐generation and even more advanced technologies today. The application of ADCs has successfully expanded from hematological malignancies to various solid tumors, including breast cancer (BC), urothelial carcinoma (UC), and lung cancer [18, 19, 20, 21].

Beyond ADCs, many other drug conjugates, represented by peptide–drug conjugates (PDCs), have rapidly gained attention as a complementary class of targeted conjugated therapeutics. In contrast to antibody carriers (∼150 kDa), peptides provide smaller size, rapid tissue penetration, flexible chemical synthesis, and tunable binding to tumor‐associated receptors, potentially enabling improved intratumoral distribution and distinct pharmacokinetic behaviors [22, 23, 24, 25]. However, PDCs also face unique hurdles, such as proteolytic instability, rapid renal clearance, and the need to balance affinity with exposure [26, 27]. The expanding repertoire of conjugate formats, including PDCs, aptamer–drug conjugates (ApDC), and other ligand‐directed systems, signals a broader shift toward modular, programmable drug delivery platforms tailored to tumor biology and clinical context.

In this review, we summarize current understandings and future perspectives of ADCs and PDCs and other drug conjugates as leading targeted conjugate modalities in oncology. We first outline the design principles shared across conjugates, target selection, carrier properties, linker technologies, payload classes, and release mechanisms, and then discuss the developmental trajectory of ADCs and the emerging landscape of PDCs. We further highlight clinical progress, safety considerations, and translational barriers, and we conclude by proposing future directions, including next‐generation conjugation chemistries, novel payloads (including immune‐modulatory agents), rational combinations with immunotherapy or DNA damage, targeting therapies, and biomarker‐driven patient selection to maximize clinical benefit.

## 2 Emergence of Concepts and Formation of Technology (Early 20th Century – 1990s)

### 2.1 The Proposal and Initial Implementation of the “Magic Bullet”

Paul Ehrlich, as the father of chemotherapy, first proposed the concept of “magic bullets” in the early 20th century. His experience in the study of infectious diseases and rich imagination led him to propose this conjecture: drugs can act directly on predetermined cellular structural targets. It means that targeted drugs should be effective against pathogens but remain harmless in healthy tissues [16]. It was not until 1958 that French oncologist George Mathé first linked an antimouse leukocyte antibody to aminopterin using a chemical linker believed to be stable, aiming to deliver the cytotoxic drug precisely to leukemia cells and improve efficacy while reducing toxicity to normal tissues. However, the linker proved unstable in vivo, causing premature drug release [28, 29]. This unstable linker has therefore attracted much attention. Despite its technical limitations, this pioneering experiment was considered one of the first in the development of ADCs.

Later, based on the efforts of predecessors, Georges Köhler and César Milstein successfully developed a method to generate permanent tissue culture cell lines capable of producing predetermined specific antibodies through cell fusion technology in 1975, known as hybridoma technology [30]. This breakthrough laid the foundation for the preparation of mAbs. Almost simultaneously, the emergence of another groundbreaking technology in the field of chemistry, solid‐phase peptide synthesis, generated significant global attention. In the 1970s, scientist Bruce Merrifield realized his brilliant idea. He built polypeptide chains by adding amino acids from the carboxyl to the amino termini through repeated deprotection and condensation reactions on insoluble solid‐phase carriers [31, 32]. This discovery earned him the 1984 Nobel Prize in Chemistry. These breakthroughs in two key technologies made ADCs truly achievable.

### 2.2 Structure Definition and First Human Trial

Before the advent of mAbs, earlier ADCs relied on polyclonal antibody conjugates, which had lower target specificity and higher potential toxicity. The shift occurred in 1983, when investigators conducted the first human trials of ADCs using mAb, using an anticarcinoembryonic antigen antibody–vindesine conjugate in eight patients with advanced metastatic cancer [33]. Although the efficacy of this treatment could not be proven in the end, the demonstrated ability to localize the tumor site proved that ADCs were theoretically feasible and also laid the foundation for subsequent technical improvements and drug optimization [34]. Since then, scientists have found that ADC consists of three primary components: a mAb, a small molecule drug, and a linker molecule [35]. The drug attached to the antibody is referred to as the “payload.”

**FIGURE 1:**
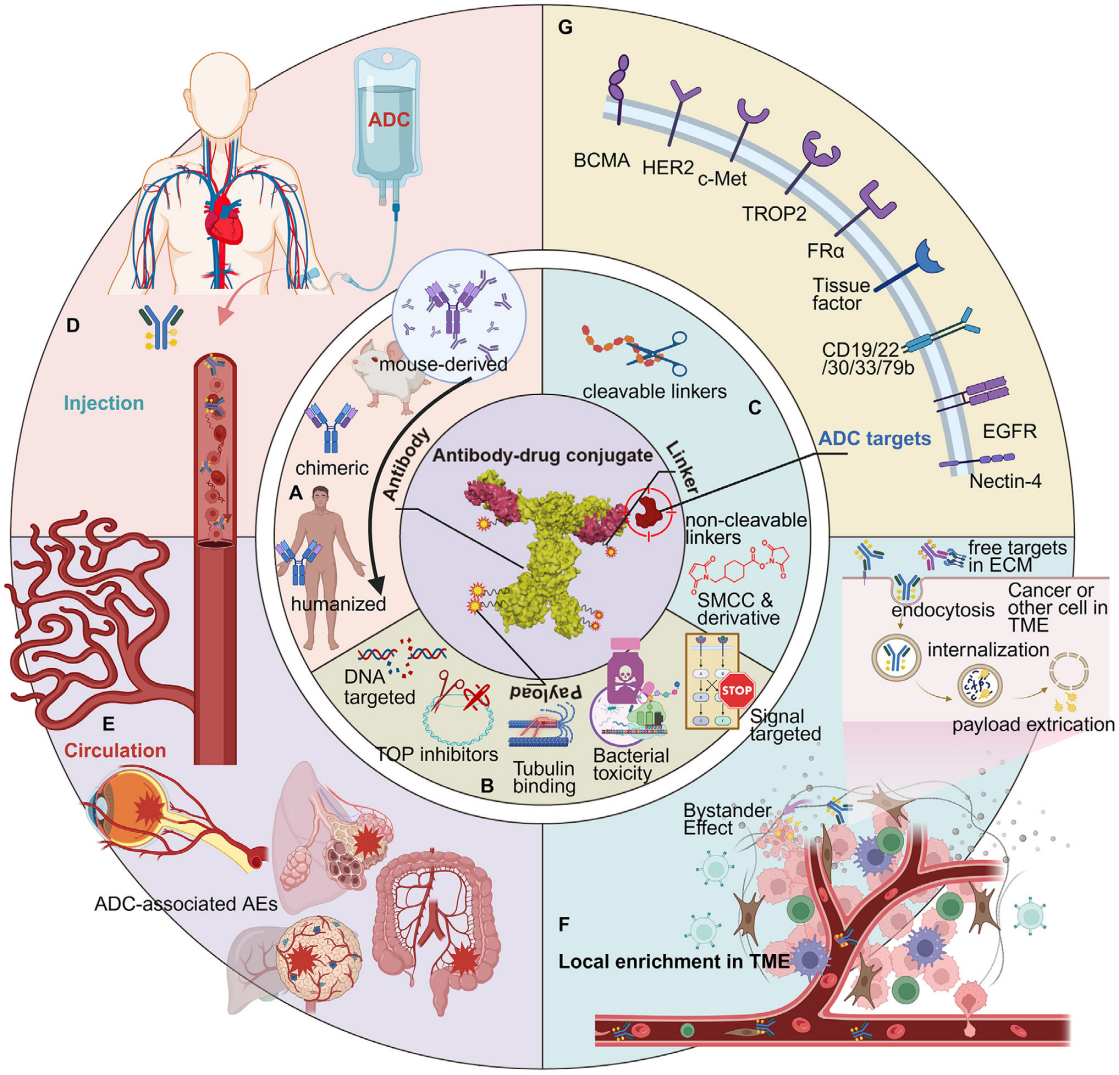
Structures and mechanism of action of ADCs. The development history of structures include: (A) antibody (mouse‐derived, chimeric, and humanized), (B) payload (tubulin inhibitor, DNA‐damaging agents, Topoisomerase I inhibitors, bacterial toxins, and photosensitizers), (C) linker (noncleavable and cleavable). The mechanism of action of ADC mainly includes some steps: (D) ADCs are administered by intravenous injection and enter the systemic circulation. (E) Systemic circulation delivers ADCs to tumors, while ADC‐associated adverse events may arise from off‐target toxicity or premature payload release. (F) The antibody component of the ADC binds to tumor‐associated antigens, leading to internalization, linker cleavage, and the intracellular release of its cytotoxic payload, which exerts a potent cell‐killing effect. Additional mechanisms within the tumor microenvironment include bystander effects (killing of neighboring antigen‐negative cells) and local enrichment via binding to free targets in the extracellular matrix. (G) ADC targets include BCMA, HER2, TROP2, EGFR, and others.

The traditional mechanism of action of ADCs mainly includes the following key steps [36, 37]. First, ADCs are administered by intravenous injection and enter the systemic circulation. Then, the mAb fraction in ADCs can specifically bind to the antigen on the surface of the target cell to form a stable antibody–antigen complex. This binding process is the basis for the accurate localization of ADCs to target cells, which ensures that ADCs can be accurately recognized and bound to the target cell surface. Subsequently, this antibody–antigen complex is internalized by target cells through receptor‐mediated endocytosis to form endocytic vesicles. Endocytic vesicles gradually mature within the cell and eventually fuse with lysosomes. After that, the linker connecting the antibody and the cytotoxic drug is cleaved by specific enzymes or the acidic environment itself, releasing the cytotoxic payload. Finally, these releasing cytotoxic drugs then enter the cytoplasm and interfere with the normal physiological functions, such as inhibiting microtubule polymerization or causing DNA damage, ultimately leading to the death of the target cell (Figure 1).

## 3 Evolution and Comprehensive Optimization of ADCs Components

**FIGURE 2:**
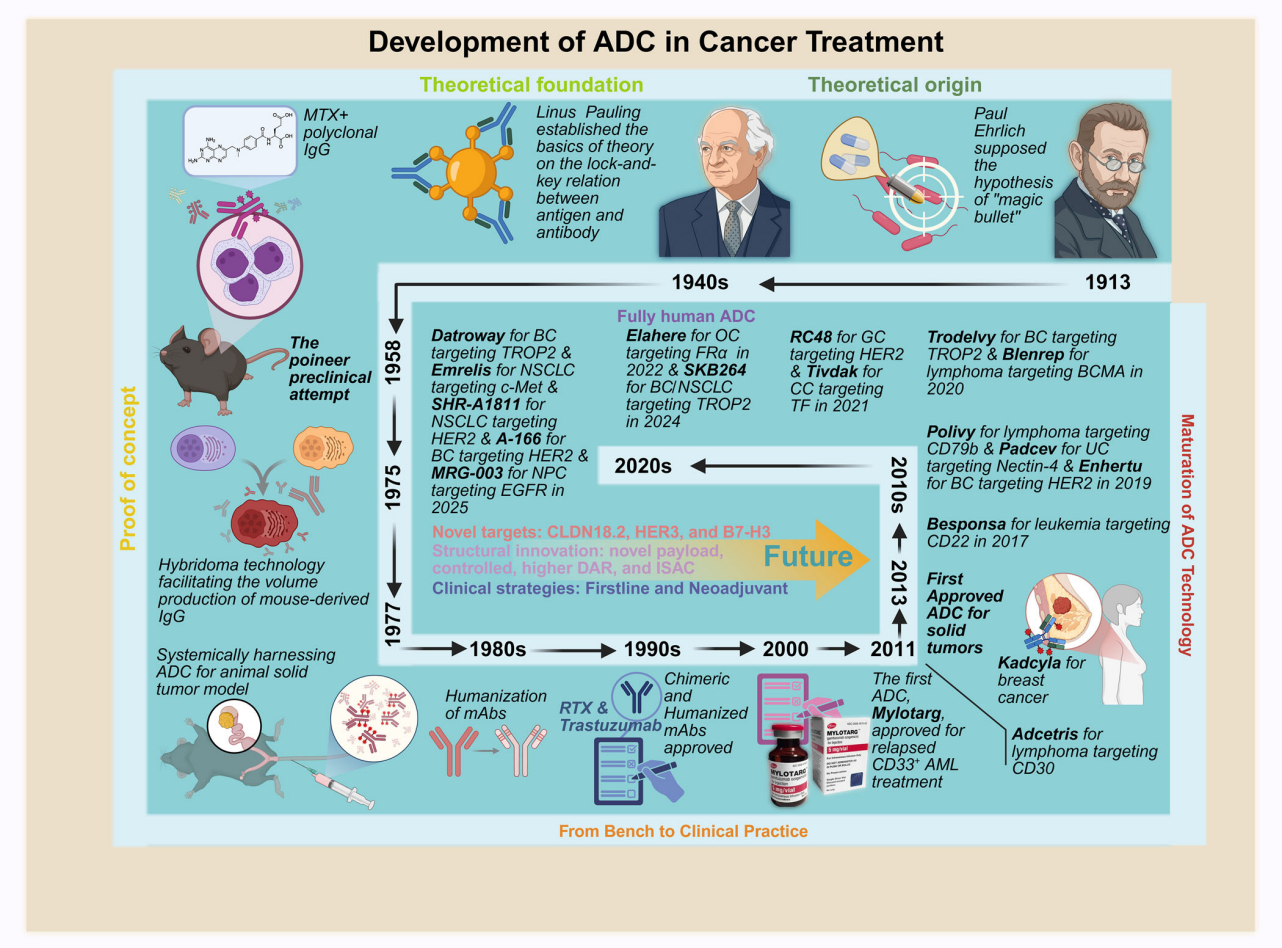
Timeline of ADC development in cancer treatment. Building upon the foundational concepts of Paul Ehrlich's “magic bullet” hypothesis and the structural insights into antigen–antibody interactions, ADCs have since embarked on their developmental journey, achieving several critical milestones along the way. These include the humanization of monoclonal antibodies, the approval of the first ADC, Mylotarg, in 2000, and the subsequent arrival of a series of drugs targeting both hematologic malignancies and solid tumors, such as Kadcyla, Enhertu, and Trodelvy. Recent and emerging directions include novel targets (CLDN18.2, HER3, B7‐H3), structural innovations (higher DAR, new payloads and linkers), and expanded clinical applications such as first‐line and neoadjuvant therapy.

Scientists have drawn certain inspiration from the use of ADCs in cancer treatment. Although the first attempt in 1983 did not yield satisfactory results, in this human trial, scientists witnessed the feasibility of future ADCs treatment, while also uncovering many problems that needed improvement, such as the accuracy and immunogenicity of mAbs, the efficacy and the therapeutic window of payload, the stability and the cutting pathways of linkers (Figure 2).

### 3.1 The Renewal of mAbs

To reduce immunogenicity, enhance tolerance and efficacy, mAbs have gradually evolved from murine origin when they were initially discovered to fully human origin at present. The first mAb approved for human use by United States Food and Drug Administration (FDA) in 1986, Muromonab CD3 (Orthoclone OKT3), was a murine mAb belonging to the IgG2a subtype that was designed to target CD3 molecules on the surface of T cells. By binding to CD3, Orthoclone OKT3 can inhibit T cells activation, thereby reducing immune rejection after transplantation [38]. Orthoclone OKT3 significantly reduces acute rejection after kidney transplantation, but patients develop antidrug antibodies after treatment, which reduces the efficacy of the drug and may cause serious side effects, such as cytokine release syndrome [39]. Unfortunately, due to these serious adverse effects during treatment, it was eventually removed from the market in 2010.

With the development of molecular biology and gene editing technology, in order to overcome the serious adverse reactions of mouse‐derived mAbs, chimeric antibodies have gradually emerged. In the 1990s, chimeric antibodies with a constant region of human immunoglobulin, including fragment crystallizable (Fc), and a variable region of mouse were developed to inhibit human antimouse antibody responses in patients. They used recombinant DNA technology to insert the light‐chain and heavy‐chain variable region of mouse mAb into the expression vector containing the constant region of human antibody, and transformed mammalian cells to express the resulting mAb [40, 41]. More importantly, engineering Fc can further optimize its binding ability to Fcγ receptors, enhance antibody‐dependent cell‐mediated cytotoxicity activity, and improve the therapeutic effect of antibody drugs [42]. This technology has led to the widespread use of mAbs in clinical practice. In 1994, the first chimeric mAb approved by the FDA for clinical use, abciximab, was used to treat cardiovascular disease [43], and in 1997, rituximab was the first FDA‐approved treatment for cancer. It is mainly used for the treatment of relapsed or refractory, CD20‐positive, B‐cell, low‐grade, or follicular non‐Hodgkin's lymphoma [44]. So far, chimeric antibodies have evolved from the initial human–mouse chimeric antibodies to a variety of forms and technology platforms, including nano‐antibody chimeras [45], bispecific/multispecific antibodies [46], multimodal targeting chimeras [47], synthetic T cell receptors [48] (such as CAR and STAR), and others. They play an increasingly important role in various disease fields, especially in tumor immunotherapy, and are continually developing in the direction of greater precision, efficiency, and safety [49].

However, human antimouse antibody responses have also been detected in patients receiving the therapeutic chimeric mAb. To minimize this reaction as much as possible, we need to further reduce the immunogenicity of the chimeric mAb by humanizing the mouse‐derived variable region [50]. One particular advance that has accelerated the development of mAbs has been the generation of humanized antibodies by means of complementarity‐determining region transplantation [51]. The first humanized mAb approved by the FDA in 1997 was the anti‐IL‐2 receptor daclizumab for the prevention of transplant rejection. The humanization of antibodies has enabled the use of novel biologic agents for the long‐term treatment of cancer and autoimmune diseases [52].

The continuous development of various technologies was also promoting the continuous optimization of antibodies, such as human antibody phage display libraries [53] and transgenic animals carrying the human antibody repertoire [54], which made fully human antibodies possible. Especially the phage display technology, which has been used for antibody maturation through site‐directed mutagenesis of complementarity‐determining region and affinity selection [55]. Based on this technology, in 2002, the first fully human therapeutic antibody approved by the FDA, adalimumab, a human antibody against tumor necrosis factor α, was used in rheumatoid arthritis [56]. To date, the FDA has approved 10 human antibody drugs produced by phage display. Chimeric or humanized antibodies have become the mainstay of antibody drugs, capable of significantly reducing immunogenicity. The advancement of mAb technology has further propelled the research and development of ADCs.

### 3.2 The Development of Linker

In the first attempt to commercialize an ADCs product, scientists discovered that a linker capable of being released in a specific environment within a cell was necessary to achieve precise payload delivery. The linker of ADCs is a key component that connects antibodies and payloads. Its structure typically consists of an antibody conjugation, an intermediate linker, and a drug linker [57]. Among them, the conjunction between the adaptor and the mAb is critical because it limits the drug–antibody ratio (DAR) and therefore affects the homogeneity and stability of ADCs [58]. It can be divided into cleavable and noncleavable. The structure of the noncleavable linker is relatively stable and does not break under physiological conditions. They generally have high chemical stability and are able to maintain ADCs integrity in the blood circulation. Upon internalization by target cells, ADCs are transported to the lysosome, where the antibody moiety is completely degraded by proteases. This process releases a cytotoxin derivative still attached to the linker and an amino acid residue. While this metabolite retains potent cytotoxicity and effectively kills the target cell, its increased hydrophilicity typically prevents efficient diffusion across the membrane, thereby limiting any significant bystander effect [58].

The advantage of cleavable linkers over noncleavable linkers is that the drug released by ADCs is membrane permeable and can exert a bystander effect. This allows a hydrophobic payload to diffuse out of the membrane of the original target cell and kill neighboring cancer cells that may not express the target antigen, with a rapid effect. This advantage plays a significant role in the treatment of solid tumors. However, it also has greater instability and is prone to off‐target toxicity. Cleavable linkers can be further divided into chemical and enzymatic cleavage linkers [57]. ADCs contain chemically cleavable linkers, including reducible linkers and acid‐cleavable linkers, which utilize the differential properties between plasma and cytoplasmic compartments to release payloads [59].

### 3.3 The Innovation in Payload

Payload, the key part of ADCs in determining the therapeutic efficacy, should ideally have high toxicity, high stability, low immunogenicity, and modifiable functional groups [60]. Among the first‐generation ADCs, chemotherapeutic agents are often used in cytotoxic payloads, such as methotrexate, doxorubicin, and vinblastine. However, due to their general lack of cytotoxicity and lack of tumor selection, killing a cancer cell often requires a large number of drug molecules. These shortcomings in the application of clinical trials have prompted an urgent need for the development of more potent cytotoxic agents. The goal of this drug is to be effective enough to kill most rapidly dividing cells without being too toxic to cause serious side effects to the patient. Subsequently, newly developed highly cytotoxic compounds were shown to be 100–1000 times more potent than the conventional chemotherapeutic drugs used in the first‐generation ADCs, which is critical because it is estimated that only 1.56% of ADCs dose will eventually reach the tumor site, and low potency will result in low intracellular drug concentrations to no effect [61].

Scientists began to search for alternative drugs, and they found that the tubulin inhibitor maytansine, a natural product extracted from plants, showed extremely potent antitumor activity. Still, its nontargeted cytotoxicity, severe neurotoxicity, and gastrointestinal effects limited its clinical application [62]. Although maytansine could not ultimately be used as an ADCs’ drug payload, it surprisingly acted as a tubulin inhibitor, inhibiting tubulin polymerization, blocking cell mitosis, arresting the cell cycle, and inducing apoptosis [63]. DM1 and DM4 are the two most commonly employed maytansine derivatives in clinical practice. Most second‐generation ADCs incorporate tubulin inhibitors as payloads. Tubulin inhibitor auristatin derivatives, including monomethyl auristatin E (MMAE) and monomethyl auristatin F (MMAF), are also widely adopted in ADCs research [64]. Meanwhile, the rapid advancement of linker and mAb technologies has facilitated the development of second‐generation ADCs.

However, in numerous studies and applications, scientists have found that antimicrotubule drugs primarily act on the mitotic link of tumor cells, but have no obvious killing effect on static tumor cells [65]. The emergence of DNA‐damaging agents has changed this dilemma by triggering intrinsic, irreversible suicide programs in cells, such as double‐strand breaks, cross‐linking, and chimerism, resulting in cells that are difficult to repair and ultimately trigger apoptosis [66]. The DNA‐damaging agents employed in ADCs including agents that cleave DNA double strands (like calicheamicins), agents that cross‐link minor grooves (like pyrrolo benzodiazepines [PBDs]) and others [67]. Topoisomerase I (TOP1) inhibitors can bind to the Topoisomerase 1 cleavage complex, thereby stabilizing it and preventing the religation of the DNA strands, leading to DNA damage, cell cycle arrest, and apoptosis [68]. Camptothecin is a naturally occurring compound that functions as a TOP1 inhibitor [69]. Several camptothecin‐derived TOP1 inhibitors, including DXd and SN‐38, have employed wildly in ADCs [67]. Emerging payloads also include bacterial toxins (such as PE38) [70] and photosensitizers (such as IR700) [71], offering new directions for the development of next‐generation ADCs. By October 2025, the FDA had approved 21 types of ADCs (Figure 2).

### 3.4 DAR: A Pivotal Force in ADCs

Although ADCs are composed of antibodies, linkers, and payloads, the therapeutic efficacy of ADCs is affected by the DAR, which affects cytotoxicity, stability, and pharmacokinetics. ADCs were initially created by nonspecifically conjugating cytotoxic medicines to antibody amino acid residues, such as lysine or cysteine, resulting in heterogeneous DAR. Even though antibodies include about 80 lysine residues, usually less than 10 of these residues can be chemically conjugated because of steric and functional limitations [72]. In practice, the average number of payloads that can be conjugated to each mAb is currently limited to 0–8 [73]. In the relatively steady state, the value of DAR is usually between 2 and 4. Only with the use of a hydrophilic linker‐payload can the DAR reach 8 [74].

Previous methods of ADCs conjugation tend to be stochastic conjugation with naturally occurring amino acids, such as lysine and cysteine, which are characterized by their abundance, high reactivity, and nucleophilic properties on native antibodies. There are dozens of lysine residues on the surface of each antibody molecule, and their side chains contain nucleophilic ɛ‐amino groups. In addition, the amino groups of lysine can be efficiently acylated with a variety of chemical groups, such as N‐hydroxy succinimide ester [75]. This conjugate method is relatively simple and easy to implement, which has significantly contributed to the development of early ADCs, such as Adcetris and Kadcyla, which utilize stochastic conjugation [76]. However, it also has disadvantages; the biggest problem is heterogeneity, which can make the ADCs’ structure unstable [77].

Subsequently, homogeneous ADCs emerged. Unlike traditional random conjugation utilizing lysines or cysteines, site‐specific conjugation involves genetic engineering of antibodies to cytotoxins at unique and defined sites. Site‐specific conjugation technology provides a powerful tool for the development and optimization of ADCs (Table 1).

**TABLE 1:** Site‐specific antibody conjugation for ADCs.

Technology	Principle	Key reagents/enzymes	Advantages	Applications/references
Engineered cysteine	Genetic engineering introduces cysteine residues at specific sites on the antibody, providing unique thiol groups for drug conjugation.	−	Achieves homogeneous DAR, improves ADCs homogeneity, stability, and therapeutic efficacy; reduces heterogeneity	Polatuzumab vedotin [78] achieve a DAR of ∼3.5 (conjugated to MMAE).
(THIOMAB)				
Disulfide rebridging	Reduces native interchain disulfide bonds to expose thiols, which are then rebridged with bifunctional linkers to conjugate drugs	Bis‐sulfones, maleimides, and their derivatives (such as N‐aryl maleimides, β‐amino maleimides)	Utilizes inherent antibody structure for site‐specific conjugation	Badescu et al. [79] first applied this for ADCs synthesis to conjugate MMAE to trastuzumab, with a DAR of 4.
Unnatural amino acids (uAAs)	Genetically incorporates uAAs at specific antibody sites, enabling bioorthogonal chemistry for precise drug linkage	p‐Acetylphenylalanine (pAcF), p‐azidophenylalanine (pAzF)	Enables precise site‐specific conjugation, resulting in homogeneous ADCs with improved efficacy, stability, and safety	1. Kularatne et al. [80] incorporated pAcF into an anti‐CXCR4 antibody and conjugated auristatin via a stable oxime bond, showing potent and selective antitumor activity.
				2. Bundy et al. [81] used pAzF and copper‐catalyzed azide‐alkyne cycloaddition (CuAAC) for direct protein conjugation.
Glycosyl modification	Utilizes the conserved N‐glycosylation site (Asn297) in the Fc region for modification, which is distant from the antigen‐binding site	Glyco‐engineering enzymes	Ideal site that does not impair antigen binding; enables homogeneous conjugation via enzymatic methods	1. Chon et al. [82] (ez‐ADiCon) prepared homogeneous anti‐HER2‐MMAE ADCs via one‐step enzymatic transglycosylation.
				2. Shivatare et al. [83] used site‐specific Fc glycan labeling to generate anti‐SSEA4‐MMAE ADCs (DAR = 4) with high internalization and cytotoxicity.
Enzymatic conjugation	Leverages the specificity and catalytic activity of enzymes to form bonds at specific tags or sequences on the antibody	Microbial transglutaminase (mTG), tyrosinase, Sortase A, etc.	Mild reaction conditions, high specificity, and excellent site‐selectivity	1. Lobba et al. [84] used tyrosinase to catalyze a bond between tyrosine and cysteine for protein–protein conjugation.
				2. Strop [85] utilized microbial transglutaminase (mTG) for site‐specific drug conjugation to generate homogeneous ADCs.

### 3.5 Site‐Specific Antibody Conjugation for ADCs

Engineered cysteine conjugation technology utilizes genetic engineering to introduce cysteine residues at specific positions of antibodies, allowing drugs to be specifically linked to these predetermined sites and achieving site‐specific conjugation. THIOMAB, developed by Junutula et al., was a special antibody that has been genetically engineered to achieve site‐specific conjugation of drugs to antibodies by introducing additional cysteine residues in the heavy or light chains of the antibody [86]. The THIOMAB–drug conjugate based on THIOMAB has been widely used in clinical practice. Polatuzumab vedotin, an ADC approved for the treatment of lymphoma, employed this technology to achieve a DAR of approximately 3.5 molecules of MMAE per antibody. This optimization improves the homogeneity and stability of ADCs while reducing heterogeneity [87, 88].

Disulfide rebridging uses the disulfide bonds present in the protein for site‐selective modification. Most proteins contain disulfide bonds that control protein stability and biological activity [89]. Disulfide rebridging involves reducing the interchain disulfide bonds in antibodies to expose sulfhydryl groups, which are then reacted with bifunctional linkers to form stable cross‐links. However, disulfide rebridging requires bifunctional reagents, and the choice of such reagents also determines the success of the conjunction. Bis‐sulfones were the initial reagents used for disulfide rebridging, and Badescu et al. first employed this method for ADCs synthesis in 2014 [79, 90]. Another widely used reagent in current applications, maleimide, exhibits distinct reactivity in Michael addition reactions with thiols, leading to the formation of thiosuccinimide. Its reactivity surpasses that of other conventional Michael acceptors, making it highly valued for its efficiency and practical utility. However, despite these advantages, maleimide is associated with a significant limitation of poor stability in practical applications [91]. The retro‐Michael reaction of traditional maleimide reagents mainly causes this instability. To solve this problem, scientists have designed N‐aryl maleimides [92], β‐amino maleimides [93], acetal containing maleimides [94], and exocyclic maleimides [95].

Different from the traditional random conjugation of natural amino acids, unnatural amino acids (uAAs) need to be genetically engineered to insert uAAs at specific positions of antibodies to obtain a uniform ADC of DAR, which has better efficacy, stability, and safety. At present, more than 50 uAAs have been genetically engineered into recombinant proteins, but *p*‐acetophenylalanine and *p*‐azidophenylalanine are the most commonly used for bioconjugate production [96]. Kularatne et al. combined the uAA *p*‐acetophenylalanine with anti‐CXCR4 antibodies through genetic engineering and linked it to the antimitotic drug auristatin via a stable oxime bond, achieving selective killing of CXCR4‐overexpressing tumor cells. At the same time, they demonstrated good efficacy and safety in both in vivo and in vitro experiments [97]. Bundy et al. achieved direct conjugation between proteins by using a direct protein click chemical conjugation method that specifically binds the azide group of *p*‐azidophenylalanine to the protein using a copper(I)‐catalyzed azide‐alkyne [3 + 2] cycloaddition reaction [81].

The glycosyl modification sites of IgG antibodies are located at residues Asn297 in the CH2 domain of the constant region. These glycosyl modifications are located far from the variable region of the antibody, making them ideal sites for chemical or biological modification of antibodies without affecting antigen‐binding properties [98]. This is also the reason why glycosylation modification sites can be specific sites for ADCs. In order to achieve glycosylation modification, various technologies have emerged. For example, Chon et al. developed a glyco‐remodeling technology called “ez‐ADiCon” that enables the preparation of homologous ADCs by one‐step enzymatic transglycosylation with payload‐preloaded biantennactin glycan complexes. They selected antihuman epidermal growth factor receptor 2 (HER2) mAb and MMAE as the model ADCs composition and verified their good cytotoxicity in vitro [82]. Shivatare et al. used site‐specific Fc glycan labeling to conjugate the chimeric anti‐SSEA4 antibody chMC813‐70 and MMAE through cleavable or noncleavable linkers, forming a homogeneous ADC with a DAR of 4. Its high internalization ability and significant cytotoxicity in SSEA‐4 positive cancer cells were also confirmed in vitro [83].

As a widely used multifunctional catalyst, enzymes are involved in almost all biochemical reactions, including metabolism, signal transduction, and immune response. It has the characteristics of mild reaction conditions, adjustability, specificity, and stability. This makes it an important tool in ADCs. Currently used enzymes are transglutaminase, Sortase A, the *E. coli* enzyme biotin ligase, and the *E. coli* lipoic acid ligase [99]. Lobba et al. achieved protein–protein and protein–peptide coupling by a bond between tyrosine and cysteine. This technique uses tyrosinase to catalyze the oxidation of tyrosine residues and the resulting intermediate reacts rapidly with cysteine residues on the target protein, resulting in site‐specific biological conjugation [84]. Strop utilized microbial transglutaminase to achieve site‐specific drug conjugation of antibodies. By introducing drugs at specific positions of antibodies, homogeneous ADCs can be generated, thereby enhancing the stability and therapeutic effect of ADCs [85].

By enhancing homogeneity and stability, optimizing pharmacokinetic profiles and therapeutic efficacy, improving the therapeutic index, and streamlining the manufacturing process, this technology significantly augments its potential for clinical application [75]. It not only advances ADCs development but also serves as a critical reference for future biotechnological applications.

## 4 Generational Evolution and Expanding Clinical Applications of ADCs

As ADCs are increasingly applied in clinical practice, many problems have emerged, such as the bottleneck of therapeutic effect, significant side effects, and easy development of drug resistance [100]. To overcome these problems, scientists have to constantly update and improve ADCs, as well as address the drawbacks that arise during the treatment process. Moreover, after these drugs have achieved good therapeutic effects, they have continuously explored and attempted to expand their treatment scope, not just limited to nonsolid tumors or solid tumors.

### 4.1 First‐Generation ADCs: Clinical Applications and Challenges

The first ADC to enter the market was used to treat acute myeloid leukemia (AML), Mylotarg (gemtuzumab ozogamicin), which is an ADC produced by conjugating calcineurin to an anti‐CD33 antibody via an acid‐labile hydrazone linker to N‐acetyl‐γ‐calicheamicin dimethyl hydrazide, a highly potent DNA‐damaging cytotoxic agent [17, 101]. It was approved by the FDA in 2000 for the treatment of relapsed CD33‐positive AML in patients older than 60 years of age who were ineligible for intensive chemotherapy. Previously, murine antibodies used in mAbs could cause severe immunogenicity. In contrast, Mylotarg uses a humanized antibody that specifically binds to the CD33 antigen, which is expressed in more than 90% of AML progenitor cells but not in normal hematopoietic stem cells. Therefore, it can selectively target malignant myeloid cells while protecting early progenitor cells and reducing the body's immune response to the drug [102]. In addition, the payload calicheamicin can produce potent cytotoxic activity at picomolar concentrations, and the availability of acid‐cleavable linkers also allows more precise drug release. However, the Phase III study conducted by the Southwest Oncology Group in the United States, S0106, showed that Mylotarg failed to demonstrate improved efficacy, and the toxicity appeared to be too high; therefore, it was voluntarily withdrawn from the market in 2010 [103].

Despite the setbacks encountered with Mylotarg, research during this period accumulated invaluable experience for ADCs technology. Scientists recognized that successful ADCs development required addressing multiple critical technical challenges, including antibody selection, linker design, cytotoxic payload optimization, and drug conjugation methodologies. These lessons laid the foundation for the subsequent advancement of second‐generation ADCs therapeutics.

### 4.2 Second‐Generation ADCs: Breakthroughs in Solid Tumors

After 2010, ADCs technology witnessed significant advancements, with second‐generation ADCs emerging one after another.

Adcetris (brentuximab vedotin), which was approved in 2011, has shown good efficacy in Hodgkin lymphoma and other CD30‐positive lymphomas. It targets CD30 and conjugates MMAE with a protease‐cleavable valine–citrulline (Val–Cit) linker, which can exert bystander effects to kill surrounding tumor cells when cleaved [104]. Therefore, Adcetris exhibits a more effective anticancer effect compared with Mylotarg. In addition, CD30, CD70, CD19, CD79b, and CD22 are also highly expressed in hematological malignancy cells, but expressed at a low level or not at all in normal cells. These alternative targets have also been used by researchers to synthesize ADCs and continuously conduct clinical trials [105].

In order to expand the therapeutic scope of ADCs, it is necessary to break through from hematological tumors to solid tumors.

In 2013, the FDA approved Kadcyla (ado‐trastuzumab emtansine, T‐DM1), jointly developed by Genentech and Immunogen, for the treatment of HER2‐positive BC [106]. It was reported that Kadcyla significantly prolonged progression‐free survival (PFS) and overall survival (OS) with less toxicity than lapatinib plus capecitabine in patients with HER2‐positive advanced BC previously treated with trastuzumab and a taxane [107]. As the first ADC targeting solid tumors, it represented a significant advancement in ADCs technology for this therapeutic area. Kadcyla incorporates a noncleavable linker to conjugate trastuzumab with the cytotoxic agent DM1 [107], preserving the targeting specificity of the antibody while enhancing drug stability and therapeutic efficacy.

In 2017, Mylotarg was reapproved by the FDA for treatment of relapsed or refractory (R/R) CD33‐positive AML in patients 2 years of age and older [108]. The reapproved regimen featured a fractionated, lower‐dose schedule (3 mg/m^2^ on Days 1, 4, and 7) compared with the initial single 9 mg/m^2^ dose [109]. This approval is based on the following three clinical trial studies: ALFA‐0701, AML‐19, and MyloFrance‐1. ALFA‐0701 is a multicenter, randomized, open‐label Phase III clinical trial enrolling 271 adult patients with newly diagnosed CD33‐positive AML [110]. Patients were randomly assigned to either the Mylotarg combination therapy group or the chemotherapy‐only group, with the primary endpoint being event‐free survival. Results demonstrated an event‐free survival of 17.3 months in the Mylotarg combination therapy group, compared with 9.5 months in the chemotherapy‐only group [111]. AML‐19 compared single‐agent Mylotarg with best supportive care including hydroxyurea as first‐line therapy in older patients with AML unsuitable for intensive chemotherapy. The result showed the median OS was 4.9 months (95% CI: 4.2–6.8 months) in the low‐dose Mylotarg group and 3.6 months (95% CI: 2.6–4.2 months) in the best supportive care group [112]. MyloFrance 1 is a single‐arm, open‐label Phase II clinical trial conducted in 57 adult AML patients in first relapse. The trial demonstrated that 15 patients achieved complete remission (CR), with a median relapse‐free survival of 11.6 months [109].

Later that same year, the FDA approved Besponsa (inotuzumab ozogamicin) for the treatment of adults with relapsed or refractory CD22‐positive B‐cell acute lymphoblastic leukemia [113]. This ADC consists of a CD22 antibody conjugated to calicheamicin, a natural product derived from Micromonospora echinospora, via an acid‐hydrolyzable butanoic acid linker. The INO‐VATE acute lymphoblastic leukemia trial (NCT01564784) demonstrated that Besponsa was superior to standard chemotherapy in achieving CR with full or partial hematologic recovery, paving a new path for patients with this aggressive and difficult‐to‐treat malignancy [114].

Lumoxiti (moxetumomab pasudotox) is also an ADC targeting CD22, generated by conjugating the recombinant murine IgG1 antibody moxetumomab to PE38 via a cleavable linker [115]. It received FDA approval in 2018 for treating adult patients with R/R hairy cell leukemia (HCL) and stands as the world's first ADC approved for HCL therapy [115]. Research indicated that Lumoxiti monotherapy achieved an objective response rate (ORR) of 75%, with a CR of 41% [116]. Among patients with HCL who had previously undergone extensive treatment, Lumoxiti demonstrated a high proportion of independently assessed durable responses alongside favorable tolerability [117].

Second‐generation ADCs primarily utilize humanized antibodies, significantly reducing immunogenicity. Regarding linkers, this stage saw the development of more stable linker technologies (such as the noncleavable linker used in Kadcyla and the enzyme‐cleavable linker in Adcetris), enhancing plasma stability and reducing off‐target toxicity. Furthermore, more potent cytotoxic drugs were introduced, such as the microtubule inhibitor MMAE/DM1, exhibiting over a hundredfold increase in efficacy compared with first‐generation agents [118]. Regarding conjugation techniques, while random conjugation remained prevalent, site‐directed engineering (such as cysteine or lysine modification) optimized DAR distribution and enhanced product homogeneity. Second‐generation ADCs have achieved significant success in both hematological malignancies and solid tumors (particularly HER2‐positive BC), demonstrating ADCs' potential as blockbuster therapies. Nevertheless, ADCs retain scope for optimization, especially for tumors with low antigen expression and heterogeneous solid tumors.

### 4.3 Rapid Advancements and Indications Expansion

From 2019 to 2021, the number of approved ADCs reached its peak. In just 3 years, a total of nine novel ADCs were approved globally. During this phase, the indications for ADCs expanded significantly. Their value was successively validated in UC, triple‐negative BC (TNBC), head and neck cancer (HNC), gastric cancer (GC), and cervical cancer, providing multiple tumor clues for ADCs applications.

The year 2019 marked a significant turning point in the development of ADCs. In that single year alone, three ADCs received approval for marketing in the United States: Polivy (developed by Roche Genentech), Padcev (developed by Seattle Genetics and Astellas), and Enhertu (developed by AstraZeneca and Daiichi Sankyo).

Polivy (polatuzumab vedotin) is the first CD79b‐directed ADC for the treatment of adult patients with R/R diffuse large B‐cell lymphoma (DLBCL), not otherwise specified, after at least two prior therapies [119]. In June 2019, Polivy received approval from the FDA based on data from the global Phase Ib/II clinical trial GO29365 (NCT02257567). In this research, the pola‐BR group (polatuzumab vedotin + bendamustine + rituximab) demonstrated significantly higher response rates than the BR group (bendamustine + rituximab) and reduced the risk of death by 58% in patients with transplantation‐ineligible R/R DLBCL. The CR rate in the pola‐BR group reached 40% (*n* = 16/40), while it was only 17.5% in the BR group (*n* = 7/40) [78]. The final analysis of the 5‐year follow‐up for the GO29365 study showed that significant survival benefit with pola‐BR versus BR persisted, with no new safety signals identified during long‐term follow‐up [120]. This result further demonstrates that Pola‐BR is an effective treatment regimen for patients with R/R DLBCL, exhibiting a well‐defined and manageable safety profile.

Padcev (enfortumab vedotin) is the first ADC targeting Nectin‐4, which can bind to cells that express Nectin‐4 with high affinity, triggering the internalization and release of MMAE in target cells [121]. Nectin‐4 is highly expressed in several solid tumors, including breast, gastric, lung, and UCs, making it an ideal target for antitumor drugs [122]. On December 18, 2019, Padcev received accelerated approval from the FDA based on data from the EV‐201 trial. In patients with locally advanced or metastatic UC who had received prior platinum and antiprogrammed death 1 or antiprogrammed death ligand 1 (PD‐1/L1) therapy, Padcev achieved an ORR of 44% (95% CI: 35.1–53.2%), a CR rate of 12% and a median duration of response of 7.6 months [123]. In the Cohort 2 of EV‐201, Padcev after PD‐1/L1 inhibitors in cisplatin‐ineligible patients with advanced UC was tolerable and confirmed responses were seen in 52% of enrolled patients (46/89) [124], which may be a promising therapy for this group of patients with limited treatment options.

Enhertu (DS‐8201, T‐DXd) is an ADC composed of a humanized IgG1 mAb targeting HER2, a TOP1 inhibitor, and an enzyme‐cleavable tetrapeptide linker (GGFG), utilizing site‐specific conjugation at cysteine residues [125]. This unique design delivers remarkable efficacy: Enhertu breaks the conventional DAR constraint of 2–4, achieving a DAR of 8, representing the maximum payload capacity for conventional interchain cysteine‐linked conjugates. A high DAR enables the drug to recognize low levels of target expression, enhancing its cytotoxic potency. In the two‐part, open‐label, single‐group, multicenter, Phase II study (DESTINY‐Breast01, NCT03248492), Enhertu (at a dose of 5.4 mg per kilogram) demonstrated durable antitumor activity in patients with HER2‐positive metastatic BC who had received extensive previous treatment, achieving a confirmed overall response rate of 60.9%, a median duration of PFS of 16.4 months, and a median response duration of 14.8 months [126]. On December 20, 2019, FDA granted accelerated approval to Enhertu for the treatment of patients with unresectable or metastatic HER2‐positive BC who have received at least two prior anti‐HER2 therapies in the metastatic setting [127]. Moreover, Enhertu has shown activity in patients with metastatic BC with low HER2 expression [128], which has heightened public attention on the bystander effect of ADCs on malignant cells beyond the target. Enhertu achieved groundbreaking success in the Destiny‐Breast series of studies, significantly improving survival and remission rates for BC patients [129]. On January 15, 2021, the FDA approved Enhertu for the treatment of patients with locally advanced or metastatic HER2‐positive gastric or gastroesophageal junction adenocarcinoma who have previously received trastuzumab therapy. According to the DESTINY‐Gastric01 (NCT03329690) study, the ORR was significantly higher in the Enhertu treatment group compared with the physician‐selected chemotherapy group (51 vs. 14%) [130]. These studies lay the foundation for the application of Enhertu in the field of pan‐cancer therapy.

In 2020, two ADCs were approved in the United States: Trodelvy from Immunomedics and Blenrep from GlaxoSmithKline. That same year, Japan's Pharmaceuticals and Medical Devices Agency also approved Rakuten Medical's ADC Akalux for marketing in Japan.

Trodelvy (sacituzumab govitecan) is a TROP2‐directed ADC, composed of the mAb sacituzumab conjugated to the toxin SN‐38 via the cleavable linker CL2A [131]. Trodelvy employs a site‐specific conjugation method, binding approximately 7–8 SN‐38 molecules to each sacituzumab antibody, with an average DAR of 7.6. CL2A is cleavable through pH sensitivity, giving rise to bystander effect, and binds the antibody at a cysteine residue via a disulfide bond. Trodelvy received accelerated approval in the United States in April 2020 for the treatment of locally advanced or metastatic TNBC (3L+), which was based on results from the Phase I/II trial (NCT01631552) [132]. Subsequent Phase III research (NCT02574455, the ASCENT study) similarly confirmed that Trodelvy provides benefits for this patient population [133]. As clinical research progresses, Trodelvy transitioned from accelerated approval to complete approval in 2021. Based on Cohort 1 of the TROPHY‐U‐01 study [134], Trodelvy was granted accelerated approval for the treatment of locally advanced or metastatic UC in patients who have previously received platinum‐based chemotherapy and checkpoint inhibitor therapy. The data from IMMU‐132‐01 basket trial further validated the safety of Trodelvy and its efficacy across multiple types of epithelial cancers [132]. Partial responses were seen in endometrial cancer (4/18, 22.2% ORR) and small‐cell lung cancer (11/62, 17.7% ORR), and one castrate‐resistant prostate cancer patient had a complete response (*n* = 1/11; 9.1% ORR). Indications for Trodelvy continue to expand.

Blenrep (belantamab mafodotin, GSK2857916) is the first in class ADC that targeted B cell maturation antigen [135]. Blenrep received FDA accelerated approval in 2020 for the monotherapy treatment of adult patients with R/R multiple myeloma who have received at least four prior therapies and whose disease is resistant to at least one proteasome inhibitor/immunomodulator/CD38 mAb and has demonstrated disease progression during the last therapy [136]. However, in November 2022, Blenrep was withdrawn from the market because of the failure of its confirmatory Phase III trial (DREAMM‐3) [137]. As the first and only ADC drug to receive FDA approval for the treatment of R/R multiple myeloma, Blenrep's clinical trial results have garnered significant attention. In 2025, two Phase III clinical trials, DREAMM‐7 [138, 139] and DREAMM‐8 [140], reaffirmed the clinical value of Blenrep. However, concerns over toxicity led to opposition against its reintroduction to the market. This served as a wake‐up call for the ADCs field: efficacy alone is not the sole measure of success. Striking a precise balance between efficacy and safety is the core logic of drug development.

Akalux (cetuximab sarotalocan sodium, ASP‐1929, RM‐1929) is a novel ADC developed by Rakuten Medical in Japan for photoimmunotherapy (PIT). PIT is an emerging tumor‐targeted phototherapy, typically referring to “near‐infrared PIT” that employs near‐infrared light irradiation [141]. It effectively integrates two cancer treatment technologies: photodynamic therapy and antibody therapy. By injecting patients with photosensitizers that bind to cancer cells and then irradiating near‐infrared light externally, the drugs trigger chemical reactions that eliminate cancer cells [142]. Akalux is a chemical conjugate of the photosensitizer IR700 with cetuximab, which targets EGFR [143]. Treatment Akalux must be used in combination with the BioBlade laser system. After the drug binds to the tumor, it can be activated locally by red laser light delivered through optical fibers. This activation triggers the release of toxins, disrupting the integrity of the tumor cell membrane and thereby killing the tumor cells. In September 2020, the Japanese government approved Akalux for the treatment of unresectable locally advanced or recurrent HNC. Researches showed that unconfirmed ORR was 43.3% and confirmed ORR was 26.7%, suggesting positive clinical outcomes [144]. The combination of Akalux and BioBlade laser systems not only precisely targets cancer but also minimizes side effects, offering a new treatment option for HNC patients.

In 2021, the FDA granted accelerated approval for two ADCs: Zynlonta and Tivdak. That same year, disitamab vedotin developed by RemeGen received marketing approval, becoming China's third‐approved ADC and the first nation domestically developed ADC.

Zynlonta (loncastuximab tesirine) is an ADC developed by ADCs Therapeutics SA, which consists of a PBD DNA‐alkylating warhead covalently attached via a cleavable linker to an anti‐CD19 antibody that binds to B cells [145]. The Phase I study showed Zynlonta had good stability in serum, notable antitumor activity, and an acceptable safety profile and the LOTIS‐2 study demonstrated that after using Zynlonta, 70 of 145 patients with R/R DLBCL after two or more multiagent systemic treatments had complete or partial response (ORR 48.3%) [146, 147]. It is approved in the United States for the treatment of R/R DLBCL in 2021 [145] and is being developed for the treatment of mantle‐cell lymphoma and follicular lymphoma [148].

Tivdak (tisotumab vedotin), originally codeveloped by Seagen and Genmab, is a first‐in‐class ADC targeting tissue factor (TF), comprising a fully human mAb specific for TF‐011 conjugated to MMAE that has been engineered to target TF expressing tumors via the protease‐sensitive cleavable linker Val–Cit [149]. The Innova201 study shows Tivdak exhibits a manageable safety profile and demonstrates encouraging preliminary antitumor activity across multiple tumor types in patients who have undergone multiple lines of prior therapy [150]. The InnovaTV204 study reveals that Tivdak achieved an ORR of 24% (95% CI: 16–33) with a median duration of response of 8.3 months [151]. Based on the results, Tivdak has been granted accelerated approval in the United States in 2021 for the treatment of adult patients with recurrent or metastatic CC with disease progression on or after chemotherapy. Results from the subsequent Phase III clinical trial (InnovaTV301) have demonstrated that among patients with recurrent or metastatic CC whose disease progressed during or after first‐line therapy, those treated with Tivdak experienced a 30% reduction in the risk of death compared with chemotherapy alone [152]. This finding exhibits significant statistical and clinical importance. In April 2024, Tivdak received full approval from the FDA.

RC48 (disitamab vedotin) is an innovative anti‐HER2 ADC, including hertuzumab (a novel anti‐HER2 mAb) coupling MMAE by a cleavable linker Val–Cit [153]. Compared with Kadcyla and Enhertu, RC48 is extremely cytotoxic at very low concentrations and the better medicinal properties of RC48 can reduce off‐target toxicity. From the Phase II study, RC48 received the ORR of 24.8% (95% CI: 17.5–33.3%) [154]. The median PFS and OS were 4.1 months (95% CI: 3.7–4.9 months) and 7.9 months (95% CI: 6.7–9.9 months), respectively. In June 2021, RC48 received its first Biologics License Application approval in China for the treatment of patients with HER2‐overexpressing (defined as IHC2+ or 3+) locally advanced or metastatic GC (including gastroesophageal junction adenocarcinoma) who have received at least two systemic chemotherapy regimens [155]. As a novel agent, RC48 as monotherapy or combination therapy is also in clinical development for the treatment of other solid cancer globally, including urothelial cancer in China and the United States, and biliary tract cancer, non‐small cell lung cancer (NSCLC) and HER2‐positive and HER2‐low expressing BC in China.

During this period, TOP1 inhibitors (such as the payload DXd for Enhertu and the payload SN‐38 for Trodelvy) emerged as star payloads. Compared with traditional microtubule inhibitors, TOP1 inhibitors exhibit low cell cycle dependency, exerting cytotoxic effects on both mitotic and nonmitotic cells. Concurrently, this payload possesses potent bystander effects, thereby overcoming tumor heterogeneity [156].

In terms of linker technology, this period saw the development of cleavable linkers that exhibit high stability in the bloodstream yet enable efficient release within the tumor microenvironment (such as the GGFG tetrapeptide‐based enzyme‐cleavable linker utilized in Enhertu). This approach minimizes off‐target toxicity while ensuring effective payload delivery at the tumor site, thereby achieving a broader therapeutic window.

### 4.4 The Era of Pan‐Solid Tumors and Combination Therapies (2022–Present)

At this stage, FDA‐approved ADCs are Elahere (2022), Datroway (2025), and Emrelis (2025). China‐approved ADCs are sacituzumab tirumotecan (2024), trastuzumab rezetecan (2025), trastuzumab botidotin (2025), and becotatug vedotin (2025).

Elahere (mirvetuximab soravtansine, MIRV) is a first‐in‐class ADC targeting folate receptor α (FRα), comprising a FRα‐binding antibody, a cleavable linker (sulfo‐SPDB), and the maytansinoid DM4, a potent tubulin‐targeting agent [157]. In the SORAYA trial of Elahere in FRα‐positive, bevacizumab‐pretreated, platinum‐resistant, high‐grade serous ovarian cancer, the investigator‐assessed ORR was 32.4% (95% CI: 23.6–42.2%) [158]. On November 14, 2022, on the basis of the positive results of the SORAYA trial, the FDA granted accelerated approval to Elahere for patients with FRα‐positive, platinum‐resistant ovarian cancer (PROC) who had previously received one to three systemic treatments. Subsequent Phase III trials demonstrated that treatment with Elahere showed a significant benefit over chemotherapy with respect to median PFS (5.62 vs. 3.98 months), OS (median, 16.46 vs. 12.75 months), and ORR (42.3 vs. 15.9%) [159]. On March 22, 2024, Elahere received complete approval from the FDA. The National Comprehensive Cancer Network guidelines recommend Elahere monotherapy as the preferred regimen for FRα‐positive PROC patients, and combination with bevacizumab as an alternative regimen for FRα‐positive PROC or platinum‐sensitive recurrent ovarian cancer.

Datroway (datopotamab deruxtecan, Dato‐DXd) is a TROP2‐directed ADC composed of a humanized mAb linked to DXd via a selectively cleavable linker, which is being developed by Daiichi Sankyo and AstraZeneca for the treatment of solid tumors [160]. The results of TROPION‐Breast01 study showed among patients with inoperable/metastatic HR+/HER2− BC, receiving Datroway had statistically significant and clinically meaningful improvement in PFS compared with investigator's choice of chemotherapy [161]. Based on the above positive results, Datroway has since been approved on January 17, 2025 in the United States for the treatment of adult patients with unresectable or metastatic HR+/HER2− BC who have received prior endocrine‐based therapy and chemotherapy. This approval offers BC patients a new treatment option and marks the ongoing advancement of ADCs in BC therapy. In TROPION‐Lung01 study, Datroway improved median PFS versus Dxd in patients with advanced/metastatic NSCLC (4.4 vs. 3.7 months) [162]. As a result, Datroway has received approval in the United States for the new indication to treat adult patients with locally advanced or metastatic EGFR‐mutated NSCLC who have previously received EGFR‐targeted therapy and platinum‐based chemotherapy.

On May 14, 2025, AbbVie announced that Emrelis (telisotuzumab vedotin, Teliso‐V, ABBV‐399) received accelerated approval from the FDA for the treatment of patients with locally advanced or metastatic, high c‐Met protein overexpressing NSCLC who have previously received systemic therapy [163]. Emrelis is the first‐in‐class c‐Met‐directed ADC with a telisotuzumab antibody, a MMAE cytotoxic payload and a cleavable MC–Val–Cit–PABC‐type linker. The pivotal Phase II LUMINOSITY trial demonstrated that the high c‐Met overexpressing group had an ORR of 34.6% (95% CI: 24.2–46.2%) and a media OS of 14.6 months (95% CI: 9.2–25.6 months) [164]. Research indicates that approximately one‐quarter of EGFR wild‐type NSCLC patients exhibit high c‐Met expression. The emergence of Emrelis offers these patients a new precision treatment option.

Sacituzumab tirumotecan (sac‐TMT, SKB264) is an ADC targeting TROP2 with three components: (1) a recombinant humanized mAb against TROP2; (2) a pyrimidine‐containing linker with a carbonate bond; (3) the TOP1 inhibitor KL610023, with a DAR of 7.4 [165]. On November 27, 2024, sacituzumab tirumotecan received marketing approval from the National Medical Products Administration for the following indication: adults with unresectable locally advanced or metastatic TNBC who have previously received at least two systemic therapies, including at least one therapy for advanced or metastatic disease. This approval is based on positive results from the OptiTROP‐Breast01 study, which demonstrated statistically significant and clinically meaningful improvements in PFS (6.7 vs. 2.5 months) and OS (not reached vs. 9.4 months) for sacituzumab tirumotecan compared with chemotherapy [166]. In patients with EGFR‐mutated advanced or metastatic NSCLC after previous treatment failure with EGFR‐tyrosine kinase inhibitors (EGFR‐TKI) and platinum based chemotherapy, sacituzumab tirumotecan also demonstrated encouraging efficacy compared with docetaxel [167]. Therefore, on March 4, 2025, sacituzumab tirumotecan received NMPA approval for its second indication: the treatment of adults with locally advanced or metastatic NSCLC harboring EGFR mutations progressed after EGFR‐TKI and platinum‐based chemotherapy. This is the global first TROP2 ADC drug approved for the indication of lung cancer. On September 30, 2025, the NMPA formally approved the third indication for sacituzumab tirumotecan for the treatment of adult patients with locally advanced or metastatic EGFR mutation‐positive NSCLC whose disease has progressed following EGFR‐TKI therapy. This approval is primarily based on the interim analysis results from the Phase III OptiTROP‐Lung04 study, which demonstrated that sacituzumab tirumotecan significantly prolonged PFS compared with platinum‐based doublet chemotherapy and achieved an improvement in OS [168]. These findings advance the application of sacituzumab tirumotecan to the forefront of treatment, offering an improved solution for patients with EGFR‐TKI resistance. Additionally, sacituzumab tirumotecan is being investigated for advanced solid tumors such as GC and gynecological malignancies, with relevant clinical trials currently underway.

Trastuzumab rezetecan (SHR‐A1811) is a novel ADC consisting of a humanized HER2‐directed mAb, cleavable tetrapeptide‐based linker, and DNA TOP1 inhibitor. It binds to HER2‐expressing tumor cells and undergoes endocytosis, where it is cleaved by proteases within the lysosomes to release toxins. This process induces cell cycle arrest, ultimately triggering tumor cell apoptosis. In a multicenter, single‐arm, Phase II trial conducted across 35 hospitals in China (HORIZON‐Lung), for 94 patients with locally advanced or metastatic NSCLC with an activating HER2 mutation, 69 (73%; 95% CI: 63.3–82.0%) of them had a confirmed objective response after treated with trastuzumab rezetecan [169]. On May 29, 2025, the NMPA announced the approval of trastuzumab rezetecan for marketing. This monotherapy is indicated for the treatment of adult patients with unresectable locally advanced or metastatic NSCLC harboring an activating HER2 (ERBB2) mutation who have previously received at least one prior systemic therapy. This achievement not only offers patients a new treatment option but also marks a milestone for China's innovative drugs in the global lung cancer ADCs field. Trastuzumab rezetecan has currently received breakthrough therapy designation from the NMPA for eight indications across the treatment areas of lung cancer, BC, colorectal cancer, GC, or adenocarcinoma of the gastroesophageal junction, biliary tract cancer, as well as epithelial ovarian cancer, fallopian tube cancer, or primary peritoneal cancer. It holds promise for benefiting a broader patient population in the future.

Trastuzumab botidotin (A166) is an innovative HER2‐directed ADC. Its structure consists of a HER2 mAb linked to Duo‐5 (a novel, highly cytotoxic MMAF derivative) via a stable protease‐cleavable Val–Cit linker, with a DAR of 2 [170, 171]. Based on the results of KL166‐III‐06 study (NCT06968585), trastuzumab botidotin was approved for marketing by the NMPA in for adult patients with unresectable or metastatic HER2 positive BC who have received one or more prior anti‐HER2 therapy on October 17, 2025. The Phase III study showed that trastuzumab botidotin significantly improved PFS compared with T‐DM1 (11.1 vs. 4.4 months; HR 0.39 [95% CI: 0.30–0.51], *p* < 0.0001) and the ORR was 76.9% with A166 versus 53.0% with Kadcyla [172]. Trastuzumab botidotin differentiates itself from Kadcyla through superior PFS, increased response rates, and a favorable tolerability profile, marking a meaningful evolution in HER2‐targeted treatment.

The 2025 American Society of Clinical Oncology featured the disclosure of results from the Phase IIb clinical trial (NCT05126719) investigating becotatug vedotin (MRG003) in patients with advanced nasopharyngeal carcinoma. Becotatug vedotin is a novel ADC of an anti‐EGFR humanized IgG1 mAb conjugated with MMAE via a Val–Cit linker [173]. As of June 30, 2024, the study results demonstrated that becotatug vedotin achieved a significantly improved ORR as assessed by blinded independent central review compared with chemotherapy (30.2 vs. 11.5%). Furthermore, PFS was also significantly improved in the becotatug vedotin arm (HR = 0.63, 95% CI: 0.43–0.91, *p* = 0.0146). The median PFS (95% CI) per blinded independent central review was 5.8 months (4.2–6.2) versus 2.8 months (2.0–5.5), respectively [174]. On October 30, 2025, the NMPA approved becotatug vedotin for recurrent or metastatic nasopharyngeal carcinoma patients who had failed platinum chemotherapy and PD‐1/L1 inhibitor.

Beyond the aforementioned marketed ADCs, among those still in clinical development, luveltamab tazevibulin (STRO‐002) has demonstrated impressive efficacy in FRα‐positive cancers. Luveltamab tazevibulin is a novel ADC that conjugates an anti‐FRα targeting antibody to a tubulin‐targeting 3‐aminophenyl hemiasterlin payload through a cathepsin‐B‐cleavable Val–Cit linker. Since this linker mixes cytotoxic drug at specific sites within the high‐affinity anti‐FRα antibody, a homogeneous ADC with a DAR of 4 was generated [175]. It has been shown to show significant antitumor activity in AML, multiple ovarian and endometrial cancer models, both as monotherapy and in combination with other agents [175, 176]. The success of the preclinical study lays the foundation for further clinical research. In a Phase I study (NCT03748186), investigators designed a modified 3+3 dose escalation study with a dose expansion in which luveltamab tazevibulin was administered intravenously every 3 weeks for ovarian or endometrial cancer. Recent data from dose expansion studies indicate that luveltamab tazevibulin demonstrates efficacy (ORR [95% CI], 37.5%) at initial doses between 4.3 and 5.2 mg/kg in patients with recurrent epithelial ovarian cancer, even when FRα expression levels are as low as TPS > 25%. These results support the continuation of clinical investigations in this patient population [177]. Therefore, to further investigate the efficacy and safety of luveltamab tazevibulin versus investigator's choice chemotherapy in women with ovarian cancer (including fallopian tube cancer or primary peritoneal cancer) expressing FRα, the same group designed a randomized, multicenter, international, open‐label, two‐stage, Phase II/III study (NCT05870748) [178]. In addition, luveltamab tazevibulin showed a strong tumor growth inhibition effect on FRα‐expressing tumors in an animal model of NSCLC, and the mechanism by which luveltamab tazevibulin activates the immune system by inducing immunogenic cell death was also revealed. Moreover, the combination therapy with immune checkpoint blockade has been proven to be more effective. These findings provide strong preclinical evidence for luveltamab tazevibulin in the treatment of NSCLC, and a Phase II study (NCT06555263) of luveltamab tazevibulin in adults with advanced or metastatic NSCLC is now underway [179].

Presently, ADCs’ targets are diversifying beyond popular targets like HER2, TROP2, and Nectin‐4. ADCs targeting novel targets such as 5T4, CD276 (also B7‐H3), CDH17, SSTR2, and PD‐L1 have also been developed [180, 181, 182, 183, 184] (Table 2). Among these, as a target that serves as both an immune checkpoint and a tumor‐associated antigen, PD‐L1 endows its ADCs with a unique dual mechanism: the targeted delivery of cytotoxic payloads coupled with the reversal of immune suppression. Preclinical studies have indicated that, compared with other approved anti‐PD‐L1 mAbs, PD‐L1‐targeting ADCs demonstrate higher cellular internalization rates, potent cytotoxic activity, and retain the potential to block the PD‐1/L1 immune checkpoint pathway [184]. HLX43 is a novel PD‐L1‐targeting ADC consisting of an engineered anti‐PD‐L1 humanized IgG1 antibody linked to a camptothecin‐based toxin [185]. In in vivo efficacy studies, HLX43 induced tumor regression in multiple PD‐L+ CDX and PDX models, and was well tolerated across all dosing groups. Based on a Phase I study, safety, tolerability, and preliminary efficacy of HLX43 in advanced/metastatic solid tumors has also been demonstrated [186]. Currently, relevant Phase II clinical trials are underway for multiple tumor types, including NSCLC (NCT07269782), cervical cancer (NCT06769152), hepatocellular carcinoma (NCT06742892), and esophageal cancer (NCT06769113). Furthermore, ADCs continue to advance toward first‐line therapy, exploring earlier treatment stages and expanded indications. They now cover the solid tumor spectrum and are extending into difficult‐to‐treat cancers.

**TABLE 2:** Some ADCs that in clinical development stages.

Target	Drug/code name	Payload	Linker	DAR	Indication	Highest clinical stage reached
5T4	ACR246 [187]	D2102	A cleavable linker	8	Advanced solid tumors	Phase I/IIa (NCT06238401)
	JK06 [188]	MMAE	A cleavable linker	2	Unresectable locally advanced or metastatic cancer	Phase I/II (NCT06667960)
CD276	BGB‐C354 [189]	A novel TOP1i payload	A cleavable linker	8	Advanced solid tumors	Phase I (NCT06422520)
	MGC026 [190]	Exatecan	A cleavable linker	/	Advanced solid tumors	Phase I (NCT06242470)
CH17	YL217 [191]	YL0010014	An enzymatically cleavable methylsulfonyl pyrimidine tripeptide drug linker	/	Advanced solid tumors	Phase I (NCT06859762)
	TAVO307 [192]	MMAE	A lysosomal cleavable dipeptide linker	4	Pancreatic, colon, and gastric cancer	Preclinical
	SOT109 [193]	Exatecan	A hydrophobic linker	/	Gastrointestinal cancers including colorectal cancer	Preclinical
SSTR2	CS5005 [183]	Exatecan	A proprietary CSL linker	/	SSTR2‐positive tumors	Preclinical
LIV‐1	BRY‐812 [194]	MMAE	CysLink	/	Advanced malignancies	Phase IIa (NCT07311538)
	SDP03923‐000‐9106 [195]	MMAE	A cleavable linker	6	Breast cancer, esophageal squamous cancer, gastric cancer, prostate cancer, and non‐small cell lung cancer	Preclinical

### 4.5 HER2‐Low Revolution

Studies have shown that patients with tumors exhibiting low HER2 expression (such as BC, GC, colorectal cancer, and UC) can benefit from anti‐HER2 ADCs, including the Enhertu. In 2022, the results of the DESTINY‐Breast04 study (NCT03734029) were released, for patients with HER2‐low (defined as a score of 1+ on immunohistochemical [IHC] analysis or as an IHC score of 2+ and negative results on in situ hybridization) metastatic BC who had received one or two previous lines of chemotherapy, the Enhertu group demonstrated a median PFS that was 4.8 months longer than the treatment of physician's choice group (9.9 vs. 5.1 months) and a 6.6‐month longer OS (23.4 vs. 16.8 months) [128]. The DESTINY‐Breast04 study results have for the first time disrupted the traditional binary classification of HER2 status in BC, marking the dawn of a new era of HER2‐based tripartite classification in BC. This advancement expands the beneficiary population for anti‐HER2 therapy beyond the traditional HER2‐positive cohort to include those with low HER2 expression, representing a historic breakthrough in the landscape of anti‐HER2 treatment for BC. Subsequently, the DESTINY‐Breast06 study further expanded the boundaries of anti‐HER2 targeted therapy [129], confirming the benefit of Enhertu in patients with low and ultra‐low HER2 expression. Results demonstrated a statistically significant and clinically meaningful improvement in PFS in the Enhertu group compared with the treatment of physician's choice group among patients with low HER2 expression, with median PFS of 13.2 and 8.1 months, respectively. The results were consistent in the exploratory HER2‐ultralow population. On January 27, 2025, DS‐8201 received renewed FDA approval for use in adult patients with unresectable or metastatic HR+ BC that is HER2‐low or HER2‐ultra‐low. The current understanding is that the mechanism of action of novel HER2 ADCs, including the bystander effect and high DAR, enables their efficacy against tumors with low or ultra‐low HER2 expression [196]. The DESTINY‐Breast15 study (NCT05950945) explored the HER2 IHC 0 population [197], potentially marking another milestone advancement in the HER2‐low expression setting by further defining the lower threshold of HER2 expression associated with Enhertu treatment benefits.

### 4.6 Combination Therapy

Multiple clinical and preclinical studies indicate that ADCs not only directly kill tumor cells through precise delivery of cytotoxic payloads but also induce immunogenic cell death and release tumor‐associated antigens [198, 199]. When combining with immunotherapies such as immune checkpoint inhibitors, T‐cell costimulatory molecules, or bispecific antibodies, ADCs significantly enhance immune activation within the tumor immune microenvironment. ADCs are evolving from a traditional “targeted drug delivery tool” into a “dual‐engine approach of targeted therapy and immune activation,” bridging precision treatment with immune regulation.

On December 15, 2023, the FDA approved Padcev in combination with pembrolizumab for the first‐line treatment of locally advanced or metastatic UC. This is the globally first approved combination therapy of a PD‐1 inhibitor and an ADC drug for treating solid tumors, making Padcev the first ADC drug approved for first‐line treatment. The EV‐302 study results indicated that compared with standard chemotherapy, Padcev plus pembrolizumab significantly extended OS (31.5 vs. 16.1 months) and PFS (12.5 vs. 6.3 months), reducing the risk of progression or death by 55% [200]. It achieved CR in nearly 29% of patients, significantly higher than the 12% rate with standard chemotherapy, while maintaining manageable safety. The patient‐reported outcomes showed that patients with moderate to severe baseline pain had clinically meaningful improvements in worst pain and GHS/QOL after combination therapy [201]. These data provide further evidence to support the use of Padcev plus pembrolizumab as a preferred treatment option for patients with previously untreated locally advanced or metastatic UC.

On June 04, 2025, the combination therapy of Trodelvy and pembrolizumab demonstrated positive results in the Phase III clinical trial ASCENT‐04/KEYNOTE‐D19 [202]. The primary endpoint, median PFS, showed a significant improvement compared with the chemotherapy plus pembrolizumab group (11.2 vs. 7.8 months), representing a significant 35% reduction in the risk of disease progression or death. This novel combination therapy holds promise as a superior option for PD‐L1‐positive TNBC patients.

TROPION‐Lung02 (NCT04526691) is a Phase Ib study designed to evaluate Datroway in combination with pembrolizumab, with or without platinum‐based chemotherapy, in patients with previously untreated or pretreated locally advanced NSCLC [203]. The latest confirmed ORR 55% in doublet (Datroway + pembrolizumab) and 56% in triplet (Datroway + pembrolizumab + Pt‐CT). These data support evaluating the efficacy of Datroway combined with immunotherapy ± platinum‐based chemotherapy versus standard treatment regimens in the ongoing pivotal Phase III studies such as TROPION‐Lung07 [204], TROPION‐Lung08 [205], and the AVANZAR study [206].

The TROPION‐Lung04 study is also a Phase Ib, 11‐cohort trial that innovatively evaluated the efficacy of Datroway in combination with rilvegostomig (a PD‐1 and TIGIT bispecific antibody) for patients with advanced or metastatic NSCLC without actionable genomic alterations [207]. Confirmed ORR for all patients was 57.5% and the disease control rate was 95.0%. Responses were observed across both squamous (45.5%; 95% CI: 16.7, 76.6) and nonsquamous histologies (62.1%; 95% CI: 42.3, 79.3) and all PD‐L1 levels. These data support the ongoing Phase III pivotal study TROPION‐Lung10 [208], which further explores the application and efficacy of Datroway in combination with rilvegostomig in patients with untreated nonsquamous NSCLC.

The ongoing Phase II OptiTROP‐Lung01 study (NCT05351788) evaluated the combination of sacituzumab tirumotecan and tagitanlimab (KL‐A167), an anti‐PD‐L1 antibody, as first‐line therapy in patients with advanced or metastatic NSCLC who lack actionable genomic alterations [209]. The results indicate that the combination therapy demonstrates good efficacy and manageable safety.

An open‐label, multicenter, Phase Ib/II study was designed to evaluate the safety and efficacy of 9MW2821 combined with Toripalimab in advanced or metastatic UC [210]. The ORR was 87.5% (35/40, 95% CI: 73.2–95.8%), including 7.5% CR rate (confirmed ORR was 80%) and the disease control rate was 92.5% (37/40, 95% CI: 79.6–98.4).

Poly (ADP‐ribose) polymerase (PARP) inhibitors are a class of drugs that interfere with PARP. By blocking DNA damage repair pathways, they have achieved breakthrough applications in treating tumors with homologous recombination deficiency through the synthetic lethality effect [211]. When ADCs kill tumor cells, the cytotoxic payloads they carry can induce DNA damage within these cells. Combining ADCs with a PARP inhibitor at this stage can further inhibit the tumor cell's ability to repair DNA damage, creating a synergistic lethality effect and enhancing tumor cell killing [212].

A preclinical study has showed that the combination of Enhertu and DNA damage response pathway inhibitors (especially olaparib, a PARP inhibitor) induced additive or synergistic antitumor effect in HER2‐low TNBC [213]. Moreover, researchers found in vitro, sequential Trodelvy followed by talazoparib (a PARP inhibitor) delayed TOP1 cleavage complex clearance, increased DNA damage, and promoted apoptosis. In the clinical trial, sequential Trodelvy/talazoparib demonstrated median PFS of 7.6 months without dose‐limiting toxicities, while concurrent dosing yielded 2.3 months PFS and multiple dose‐limiting toxicities including severe myelosuppression in patients with metastatic TNBC [214].

For ovarian cancer, PARP inhibitors have greatly improved the prognosis, but drug resistance poses a significant challenge in clinical application. In olaparib‐resistance ovarian cancer models, the combination of Trodelvy with either PARP inhibitors can overcome olaparib resistance and synergistically resulted in induction of apoptosis in cell lines [215].

In summary, combination therapy for ADCs and different types of antitumor drugs not only enhance clinical efficacy but also demonstrate significant potential in overcoming the challenge of drug resistance.

## 5 New ADCs Configuration

Emerging novel ADCs configurations in recent years include bispecific ADCs, conditionally active ADCs, immunostimulatory antibody conjugates (ISACs), protein degradation ADCs, and dual‐drug ADCs. Bispecific ADCs and dual‐drug ADCs address drug resistance and tumor heterogeneity by enhancing activity, while immune‐stimulating ADCs and protein degradation ADCs achieve multimodal cancer treatment by introducing distinct mechanisms of action [216].

Bispecific ADCs are therapeutic agents composed of an antibody with two distinct antigen‐binding sites (also called bispecific antibodies) conjugated to one or more cytotoxic payload [217]. They can binding to two distinct epitopes on either the same or different antigens. By leveraging the synergistic effects of targeting two different antigens, these agents achieve more precise targeting of tumor cells, further enhancing tumor selectivity and endocytosis efficiency, and improving responses in patients with low antigen expression or heterogeneous tumors [218]. Furthermore, precise control over the antibody‐to‐toxin ratio and linkage configuration enables optimization of both safety and efficacy. Dual‐antibody ADCs broadly fall into two categories: dual‐epitope ADCs target different epitopes of the same antigen, such as JSKN003 [219], ZW49 [220], and TQB2102 [221]; the other targets two distinct antigens, known as dual‐target ADCs, including BL‐B01D1 [222], IBI3001 [223], AZD9592 [224], and IM2902 [225].

Dual‐drug ADCs conjugate two distinct payloads to a single antibody so that they can overcome resistance factors, achieve multimechanism synergy, and expand indications [226]. Based on the site of payload‐antibody linkage, they are categorized into single‐site (including tandem and parallel configurations) and double‐site (primarily engineered, unnatural AA, glycan, and microbial transglutaminase) approaches, which introduce two payloads through a two‐step process [227]. In 2017, Levengood et al. disclosed the first dual‐payload ADC, which contained two distinct microtubule‐associated protein polymerization inhibitors (MMAE and MMAF), conjugated to a CD30 antibody [228]. This design leveraged complementary anticancer activities, demonstrating significant therapeutic efficacy in a xenograft model of anaplastic large cell lymphoma resistant to monotherapy. Currently, some dual‐payload ADCs have entered clinical trials for cancer treatment, with dozens of candidates in preclinical development [229, 230, 231]. As the first dual‐payload ADCs advance into clinical studies, human data will soon emerge. Phase I trials remain primarily focused on safety. Toxicity profiles vary across different payload combinations, necessitating close monitoring of their additive effects. Additionally, efficacy metrics such as response rates and durability are equally critical. Future development and application of dual‐payload ADCs will require further data support.

ISACs, as a new generation of ADCs, possess dual therapeutic effects in eliminating tumor cells by activating both innate and adaptive immune responses [232]. This innovative technology utilizes antibodies as carriers to deliver immune agonists or modulators into the tumor microenvironment, overcoming inherent challenges associated with systemic administration of immunostimulants [233]. It holds promise for enhancing antitumor efficacy while reducing systemic side effects. The anti‐HER2 TLR8 ISAC SBT6050, also known as pertuzumab zuvotolimod, consists of a TLR8 agonist conjugated to pertuzumab via a cleavable linker [234]. In Phase I studies (NCT04460456), it was tested as monotherapy and in combination with anti‐PD‐1 antibodies pembrolizumab or cemiplimab [235]. Phase I/II studies (NCT05091528) evaluated combinations with other HER2‐targeted therapies. However, these trials were ultimately terminated due to cytokine‐related adverse events and insufficient monotherapy activity. ISAC development remains at a very early stage. While the concept is clear, overcoming the dose escalation phase in clinical trials proves challenging. Agonists targeting TLRs, STING, and similar pathways often face narrow therapeutic windows and systemic toxicity issues. As efficacy increases, toxicity also escalates. Compared with ADCs, the therapeutic window for ISACs is significantly more difficult to manage. Recently, researches developed a new ISAC called Y101S, which integrates an anti‐TGF‑β and PD‑L1 bispecific antibody with a cleavable STING agonist payload [236]. By cotargeting the TGF‑β, PD‑L1, and STING pathways simultaneously, this ISAC provides strategic benefits compared with traditional multidrug regimens and may enhance cancer therapeutic outcomes.

## 6 Peptide–Drug Conjugates

Drug conjugates have emerged as a promising drug delivery system designed to deliver payloads directly to target cancer cells, maximizing therapeutic efficacy while minimizing toxicity. Among these, ADCs have achieved remarkable efficacy in clinical applications, but they also present challenges such as the emergence of drug resistance, complex pharmacokinetics, high costs, low cellular permeability, and adverse immunotoxicity [237]. Similarly to ADCs, PDCs are defined as drugs that covalently link a drug to a peptide sequence via a specific linker. They consist of three components: a homing peptide, a linker, and a cytotoxic payload, and similarly enable the delivery of specific payloads to target tumor cells [238].

### 6.1 Comparative Analysis of PDCs and ADCs

While PDCs share a similar three‐component architecture with ADCs, they possess distinct differences that confer unique advantages and limitations [239] (Table 3). First, PDCs have a relatively low molecular weight, exhibiting strong vascular, tissue, and cellular penetration capabilities, while also demonstrating higher distribution within tumor stroma [23]. Second, regarding pharmacokinetics, PDCs are rapidly excreted via the kidneys and exhibits lower toxicity to the liver and bone marrow [240]. Third, different from the potential adverse immunotoxicity of ADCs, peptides of PDCs exhibit high biodegradability and low immunogenicity, inducing minimal immune responses in patients. Then, the short peptide of PDCs facilitate easier modification and conjugation, enabling its coupling with various drugs, which significantly enhances the technology feasibility. Furthermore, compared with mAbs, peptides are easier to synthesize and purify, making PDCs more cost effective in production, transportation, and storage. Last but not least, for payload selection, studies indicate that only a small fraction of payloads (approximately 0.1%) can reach tumor tissue when using ADCs [61]. This implies that more cytotoxic payloads are required to achieve therapeutic efficacy. In contrast, the superior tumor tissue permeability and high concentrations at the target site of PDCs expand the range of payloads available. Clinically established chemotherapy agents with relatively lower toxicity, such as doxorubicin and paclitaxel, can serve as payloads for PDCs, which also accommodate higher drug loading capacities [241]. Based on these advantages, PDCs are considered the next generation of anticancer drugs following ADCs therapeutics.

**TABLE 3:** Similarities and differences between ADCs and PDCs.

Aspect	ADCs	PDCs
Basic structure	Consist of an antibody, a linker, and a payload	Consist of peptides, a linker, and a payload
Mechanism of action	Aim to enhance efficacy and reduce systemic toxicity through targeted delivery.	
Molecular size/weight	Large molecule (typically >150 kDa) [14]	Small molecule (typically ∼2–20 kDa) [242]
Tissue/cellular penetration	Relatively low, limited penetration into solid tumors [241]	High, with excellent vascular, tissue, and cellular penetration capabilities [243]
Tumor distribution	Relatively low distribution in tumor stroma	Higher distribution within the tumor stroma
Pharmacokinetics	Long circulation half‐life (days to weeks), potential toxicity to the liver and bone marrow [241]	Short circulation half‐life, rapidly excreted via the kidneys [240]
Immunogenicity	Risk of immunogenicity, potential for antidrug antibody development [244]	Low immunogenicity, high biodegradability, minimal risk of immune response [245]
Production technology	Complex (mammalian cell culture, intricate purification processes)	Relatively simple, easier synthesis, modification, and purification, cost effective
Payload requirements	Requires highly potent payloads (due to low delivery efficiency, only ∼0.1% reaches the tumor) [61]	Broader range of payload options, can use established chemotherapeutic agents with lower toxicity (such as doxorubicin, paclitaxel) [241]
Manufacturing and cost	Complex production (mammalian cell culture, difficult purification), high cost [240]	Simple synthesis (solid‐phase peptide synthesis), easy modification, cost effective [240]

### 6.2 Structure of PDCs

#### 6.2.1 Peptide

The development of PDC's homing peptides has advanced rapidly due to technologies such as proteomics, phage display, mRNA display, and solid‐phase peptide synthesis. According to the cellular function, peptides in PDCs can be divided into cell targeting peptides (CTPs) and cell penetration peptides (CPPs).

CTPs mediate the precise delivery of drug molecules to target cells by binding to specific receptors overexpressed on the surface of tumor cells [246]. Tumor targeting peptide as an important class of CTPs are used as targeting agents for cancer therapy.

Integrins are heterodimeric receptors composed of α and β subunits, responsible for adhesion between cells, within cells, and to the extracellular matrix [247]. αvβ3 is a critical family member of integrins and interacts with extracellular matrix proteins with Arg–Gly–Asp (RGD) tripeptide sequences, indicating their potential for use in targeted cancer therapy [248]. Overexpression of αvβ3 has been demonstrated to be associated with numerous cancers, including glioblastoma [249], melanoma [250], and BC [251]. RGD peptides are widely used in targeted drug delivery. For glioblastoma, a PDC (RGD‐PEG‐Suc‐PD0325901, W22) using a combination of PD0325901 (a MEK1/2 inhibitor) and RGD peptide showed great superiority in xenografted tumors and strong antitumor efficacy [252]. A PDC composed of the RGD peptide, assembly motif (GNNNQNY), and CPT molecule can form nanoclusters to improve cell‐entry efficiency and treatment efficacy [253]. RGD‐based self‐assembling nanodrugs are a promising advancement in PDC development [254]. Except for αvβ3, the RGD peptide also exhibits high affinity for integrin αvβ6. Spirogen Ltd developed the αvβ6‐specific PDC SG3299, composed of the DNA‐binding PBD‐based compound tesirine conjugated to the αvβ6‐targeting peptide A20FMDV2 [255]. The results of animal experiments indicated that SG3299 showed efficacy against both αvβ6‐positive primary and αvβ6‐positive secondary tumors in immunocompetent animals, providing a molecular‐specific drug for the effective therapy of pancreatic cancer [256].

In addition to the aforementioned RGD peptides targeting integrins, PDC's tumor‐targeting peptides also include hormone‐associated targets (such as the SST2 receptor and GnRH receptor) and tumor microenvironment targets (such as Matrix metalloproteinase), among others. Furthermore, blood–brain barrier (BBB) targets (such as low‐density lipoprotein receptor‐related protein 1) have garnered significant attention in PDC development targeting brain metastases.

The BBB exhibits high selectivity, permitting only certain metabolically essential molecules (such as glucose, insulin, and growth hormone) and lipid‐soluble small molecules to enter the brain [257]. This property poses a significant constraint in developing effective drugs for treating brain and central nervous system disorders. ANG1005 is a novel paclitaxel–Angiopep‐2 conjugate. Angiopep‐2 is a short peptide composed of 19 amino acids that binds to low‐density lipoprotein receptor‐related protein 1 with high affinity and utilizes receptor‐mediated endocytosis to cross the BBB. Studies demonstrated that both in vitro and in vivo in human xenografts, ANG1005 had activity on brain tumors [258]. A multicenter, open‐label Phase II study (NCT01480583) in patients with measurable recurrent brain metastases from BC found 77% (intracranial) and 86% (extracranial) of patients achieved a response (disease stabilization or better) [259]. The Phase III clinical trial protocol has been approved by the FDA and is being conducted simultaneously in the United States and Canada. The preliminary success of this drug demonstrates that PDC delivery has become a promising central nervous system drug delivery system.

CPPs consist of 5–30 amino acid residues and can penetrate tissue and cell membranes via energy‐dependent or nondependent mechanisms without interacting with specific receptors, thereby effectively delivering compounds or drugs into impermeable cells [260]. The mechanisms by which CPP penetrates cell membranes can be categorized into two types: direct penetration (or “membrane transduction”) and endocytosis. Based on the properties of CPP, PDCs containing CPP offer the following advantages: (1) enhancing drug solubility and toxicity, (2) altering drug cellular penetration mechanisms to overcome resistance, and (3) reducing nonspecific drug toxicity [261].

#### 6.2.2 Linker

Similar to ADCs, linkers of PDCs can be broadly categorized into two types: noncleavable linkers and cleavable linkers. Cleavable linkers can be further subdivided based on their cleavage mechanisms: (1) Acid‐sensitive linkers: hydrolyzed under the acidic conditions of the acidic tumor microenvironment (pH 6.5–6.9) or in the acidic cellular compartments, endosomes (pH 5.5–6.2) and lysosomes (pH 4.5–5.0); (2) redox‐sensitive linkers: cleave in the reducing environment of high glutathione concentrations within tumor cells, that is, reducible disulfide linkers; (3) enzyme‐sensitive linkers: cleavable by specific enzymes overexpressed in tumor cells [262]. Upon reaching tumor cells, cleavable linkers respond to specific intracellular enzymes or microenvironmental conditions to break, enabling precise drug molecule release. Noncleavable linkers remain inactive without external stimuli, enhancing circulation stability, examples include succinimidyl thioether, oxime, and triazole [262].

#### 6.2.3 Payload

The payload of PDCs typically consists of highly cytotoxic small‐molecule compounds such as adriamycin, paclitaxel, and Zoloft Melphalan [239]. The advantage of conjugating traditional cytotoxic drugs with peptides lies in modifying their chemical properties, including solubility, selectivity, and half‐life, significantly enhancing drug bioavailability and thereby improving the efficacy of conventional therapies [263]. Furthermore, by reducing drug distribution in nontarget tissues, systemic toxicity and adverse reactions are effectively minimized. Beyond chemotherapeutic agents, other drug types can also be engineered for peptide conjugation, such as radiotracers 177Lu [264], HDAC inhibitor [265], antibodies [266], and therapeutic nucleic acids [267]. This diversity of payloads broadens the application scope of peptide‐based therapies and advances the potential for clinical translation.

### 6.3 Clinical Application of PDCs

As of October 2025, only three PDCs have been successfully approved for clinical use by FDA. Lutathera (177Lu‐Dotatate) is the world's first approved PDC and also the first FDA‐approved peptide receptor‐radionuclide therapy [264]. Lutathera combines the radionuclide 177Lu with the somatostatin analogue DOTA‐TATE, so that targeted delivery of the radioactive isotope 177Lu can be achieved. Upon entering cells, 177Lu induces DNA double‐strand breaks by emitting high‐energy β rays, ultimately leading to tumor cell apoptosis and delivering significant antitumor effects. In 2018, Lutathera was approved for the treatment of gastroenteropancreatic neuroendocrine tumors (GEP‐NETs). The Phase III NETTER‐1 study (NCT01578239) showed that the estimated rate of PFS at month 20 was 65.2% in the Lutathera group and 10.8% in the control group [268]. The secondary endpoint of OS was not met: median OS was 48.0 months in the Lutathera group and 36.3 months in the control group (two‐sided *p* = 0.30). Although OS did not reach statistical significance, the median OS in the Lutathera group was extended by 11.7 months, which may be clinically meaningful. After that, the NETTER‐2 study (NCT03972488) demonstrated the efficacy and safety of first‐line Lutathera treatment for patients with higher Grade 2–3, well‐differentiated, advanced GEP‐NETs for the first time [269]. The study findings fill a gap in the standard first‐line treatment regimen for patients with advanced high‐grade G2/G3 GEP‐NETs and will transform future clinical practice for G2/G3 GEP‐NETs.

Pepaxto (Melflufen) is a novel lipophilic PDC specifically targeting aminopeptidase, which can rapidly cross the cell membrane and utilize peptidases and esterases to release entrapped hydrophilic metabolites with alkylating activity inside tumor cells [270]. Like other nitrogen mustard drugs, Pepaxto exerts antitumor activity through DNA crosslinking. In 2021, based on results from a Phase II HORIZON trial (NCT02963493), the drug received FDA accelerated approval for the treatment of relapsed/refractory multiple myeloma [271, 272]. However, in subsequent Phase III confirmatory clinical trials (NCT03151811), the results failed to meet expectations and simultaneously revealed safety concerns, including severe hematologic toxicity [273]. Based on these clinical data, the FDA revoked the marketing authorization in 2024.

Pluvicto (177Lu‐PSMA‐617) is a radioligand therapy primarily indicated for the treatment of PSMA‐positive metastatic castration‐resistant prostate cancer who have previously received other therapy options (such as inhibition of the androgen receptor pathway and taxane‐based chemotherapy) [274, 275]. It received FDA approval for marketing in 2022. Its mechanism of action involves the conjugation of PSMA‐targeting peptides with the radionuclide Lu177 to achieve precise tumor‐targeted therapy. This delivers the radioactive source directly to prostate cancer cells for targeted radiation, sparing surrounding healthy cells. Recently, based on the positive results of the Phase III PSMAfore study (NCT04689828), the FDA approved Pluvicto for use in PSMA‐positive patients with metastatic castration‐resistant prostate cancer who have received one androgen receptor pathway inhibitor and are considered suitable for delayed chemotherapy [276].

Currently, researchers are exploring the use of Pluvicto in earlier stages of the disease, including metastatic hormone‐sensitive prostate cancer (PSMAddition, NCT04720157) and oligometastatic prostate cancer (PSMA‐DC, NCT05939414).

Additionally, a large number of PDC candidates are currently in clinical development, demonstrating significant therapeutic potential in treating various solid tumors. Their indications encompass multiple malignant tumors, including GC, liver cancer, prostate cancer, NSCLC, and BC (Table 4).

**TABLE 4:** PDCs under clinical investigation.

PDC	Target	Peptide	Payload	Indication	Clinical phase	Trial number
SNG1005/ANG1005/GRN1005 [259]	LRP1	Angiopep‐2	Paclitaxel	Measurable recurrent brain metastases from breast cancer	Phase III	NCT03613181
CBP‐1008 [277]	FRα and TRPV6	∖	MMAE	Platinum‐resistant ovarian cancer	Phase II	CTR20222941
CBP‐1018 [278]	PSMA and FRα	∖	MMAE	Advanced solid tumors	Phase I	NCT04928612
BT1718 [279]	MT1‐MMP	Bicyclic peptide	DM1	Advanced solid tumors	Phase I/IIa	NCT03486730
BT5528 [280]	EphA2	Bicyclic peptide	MMAE	Advanced solid tumors historically known for expression of EphA2	Phase I/II	NCT04180371
BT8009 [281]	Nectin‐4	Bicyclic peptide	MMAE	Locally advanced or metastatic urothelial cancer	Phase II/III	NCT06225596
TH1902 [282]	SORT1	TH19P02	Docetaxel	Recurrent advanced solid tumors	Phase I	NCT04706962
CBX‐12 [283]	∖	pH‐low insertion peptide (pHLIP)	Exatecan	Platinum resistant or refractory ovarian cancer; metastatic chemotherapy‐refractory microsatellite stable colorectal cancer	Phase II	NCT06315491;NCT06730100
DTS‐108 [284]	∖	a cationic peptide (Vectocell)	SN38	Advanced malignancies	Phase I	/
AVA‐6000 [285]	TME	∖	Doxorubicin	Locally advanced (unresectable) or metastatic solid tumors that are likely to be FAP positive	Phase I	NCT04969835
SC‐101 [286]	Nectin‐4	∖	MMAE	Locally advanced or metastatic malignant tumors who are positive for NECTIN4 gene amplification	Phase IIa	NCT07080619
BGC0228	CD44	∖	TOP1 inhibitors	Advanced solid tumors	Phase I	CTR20220194
BGC0222 [287]	αvβ3	cRGD	Irinotecan	Advanced solid tumors	Phase I	CTR20221496
AEZS‐108/AN152 [288]	LHRH	[D‐Lys6]LHRH	Doxorubicin	Endometrial cancer resistant to platinum/taxane‐based chemotherapy	Phase III	NCT01767155
L377202 [289]	PSA	Prostate‐specific antigen (PSA) peptide	Doxorubicin	Hormone refractory prostate cancer	Phase I/II	NCT00987753
PEN221 [290]	SSTR2	Tyr3–octreotate	DM1	Somatostatin receptor 2 expressing advanced cancers, including gastroenteropancreatic or lung or thymus or other neuroendocrine tumors or small cell lung cancer or large cell neuroendocrine carcinoma of the lung	Phase I/IIa	NCT02936323
G202/Mipsagargin [291]	PSMA	PSMA‐specific peptide	12ADT	Hepatocellular carcinoma; recurrent or progressive glioblastoma; clear cell renal cell carcinoma	Phase II	NCT01777594;NCT02067156;NCT02607553
MB1707 [292]	CXCR4	CXCR4 peptide antagonist	Paclitaxel	Advanced cancers	Phase I	NCT05465590
EP‐100 [293, 294]	LHRH Receptor	Membrane‐disrupting peptide CLIP 71	LHRH natural ligand	Refractory or recurrent ovarian cancer	Phase II	NCT01485848

Partial data sources: ClinicalTrials.gov (https://clinicaltrials.gov/) and the Center for Drug Evaluation, National Medical Products Administration (http://cde.org.cn/).

To date, PDCs have demonstrated promising potential in tumor therapy and broader clinical applications. Nevertheless, PDCs also face certain challenges. Peptides are rapidly degraded by proteases in the bloodstream and readily cleared by the kidneys, resulting in low circulatory stability for PDCs [295]. Moving forward, enhancing PDC stability and extending their half‐life will become key research directions, achievable through approaches such as using bicycle toxin [281] and nanoparticles technology [296]. With the broader development and application of PDCs, it is anticipated that patients will gain access to more treatment options.

## 7 Beyond ADCs and PDCs: Other Novel Conjugated Drug Platforms

**FIGURE 3:**
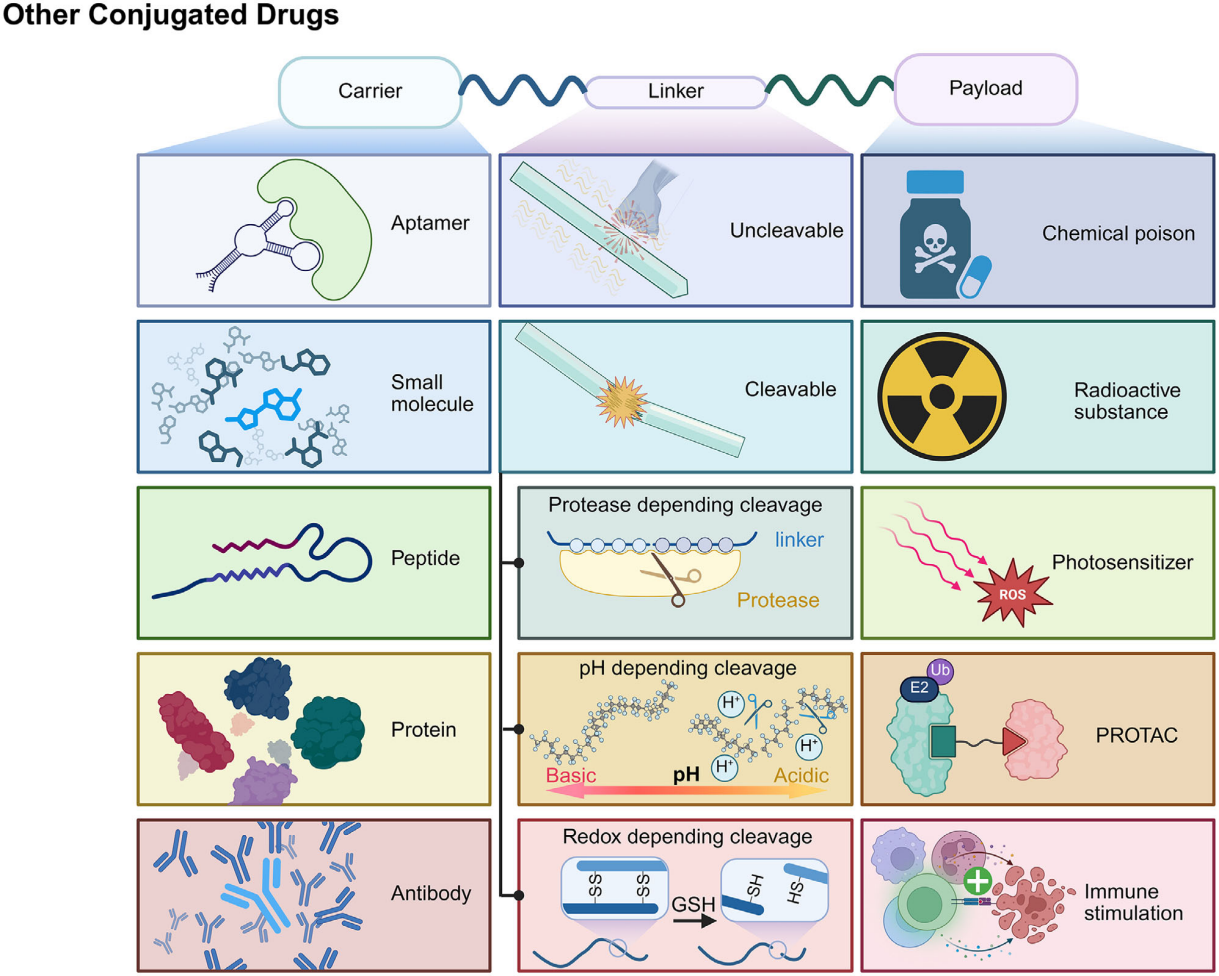
Schematic representation of other novel conjugated drugs. The architecture of conjugated drugs comprises three core elements: the carrier (such as aptamer, small molecules, peptides, protein, and antibody), the linker (including noncleavable types as well as cleavable types such as protease‐depending, pH‐depending, and redox‐depending linkers), the payload (which may be chemical poison, radioactive substance, photosensitizer, PROTAC, or immune stimulation).

In addition to the well‐established ADCs and the emerging PDCs, the field of targeted drug delivery has witnessed the rapid development of numerous other conjugated drug platforms. These next‐generation modalities leverage diverse targeting ligands (including aptamers, small molecules, nanobodies (Nbs), and even whole cells) to overcome the specific limitations of ADCs and PDCs, such as poor solid tumor penetration, immunogenicity, or limited payload diversity. They share a common principle: the use of a molecular or cellular vehicle to precisely deliver a therapeutic or diagnostic agent to a disease site [297]. These novel platforms can be categorized according to the chemical nature of their targeting ligands and their functional mechanisms. This section first presents a classification overview, followed by in‐depth discussions of nucleic acid‐based conjugates, small molecule and peptide‐derived conjugates, and next‐generation functional conjugates that integrate advanced therapeutic or diagnostic modalities (Figure 3).

### 7.1 Classification And Overview of Novel Conjugated Drugs

Based on the core targeting component and mechanism of action, novel conjugated drug platforms fall into three major families: (1) nucleic acid‐based conjugates, which use oligonucleotides (including aptamers) as targeting or therapeutic modules, with representative examples including ApDCs and antibody–oligonucleotide conjugates (AOCs); (2) small molecule and peptide‑derived conjugates, which employ low‐molecular‐weight ligands (such as folate or PSMA inhibitors) or small protein scaffolds (such as Nbs) for enhanced tissue penetration and reduced immunogenicity, this category including small molecule–drug conjugates (SMDCs) and Nb–drug conjugates (NDCs); (3) next‑generation functional conjugates, which combine antibodies or cells with emerging therapeutic paradigms like targeted protein degradation, radionuclide therapy, cell‑based immunotherapy, or biopolymer‑mediated delivery, with key examples being degrader–antibody conjugates (DACs), radionuclide–drug conjugates (RDCs), antibody–cell conjugates (ACCs), and antibody–biopolymer conjugates (ABCs).

### 7.2 Nucleic Acid‐Based Conjugates

#### 7.2.1 Antibody–Oligonucleotide Conjugate

Oligonucleotides are a class of single‐ or double‐stranded small synthetic nucleic acid polymers (approximately 20‐mer) that can be used to modulate gene expression [298]. The introduction of therapeutic oligonucleotides has expanded the scope and boundaries of disease treatment, bringing new hope for the management of numerous conditions. Since the action of oligonucleotides requires high complementarity with the target sequence, oligonucleotides are thought to be much more specific than small molecule drugs [299]. AOCs can combine the high precision of oligonucleotides with the deliverability of antibodies, thus synergizing the advantages of both technologies [300]. AOCs were initially used as diagnostic tools, but over the past decade they have gradually evolved into a novel therapeutic approach. This field remains in its infancy, with substantial preclinical research still required.

#### 7.2.2 Aptamer–Drug Conjugates

Aptamers are single‐stranded oligonucleotides (RNA or DNA) selected through systematic evolution of ligands by exponential enrichment [301]. They fold into specific structures to bind target proteins, inhibiting protein–protein interactions and thereby achieving therapeutic effects. They are also known as “chemical antibodies,” exhibiting specificity and affinity equivalent to or superior to antibodies. Compared with antibodies, aptamers offer advantages such as ease of synthesis, ease of chemical modification, high stability, and low cost [302, 303]. Therefore, aptamers show great promise in cancer treatment and diagnosis. In 2009, the concept of ApDCs was first proposed [304]. Typical ApDCs are composed of three parts: aptamer ligands, drug moieties, and linkers between aptamers and the drug moieties. ApDCs have a wide range of applications, including TNBC [305], GC [306], esophageal cancer [307], and pancreatic cancer [308]. Drugs in development include APTA‐12, APTA‐16, OLI‐0101, OLI‐0201, AST‐201, AST‐202, and others, primarily remaining in preclinical research. Among these, AST‐201 entered Phase I clinical trials on March 11, 2025 (NCT06687941). Recently, Chinese researchers developed an ApDC targeting protein tyrosine kinase 7, named Sgc8c‐M [309]. This study performs a comprehensive evaluation from rodents to nonhuman primates of Sgc8c‐M, which lays the foundation for elucidating the characteristics of ApDCs and advancing their clinical translation.

### 7.3 Small Molecule and Peptide‑Derived Conjugates

#### 7.3.1 Small Molecule–Drug Conjugates

Similar to ADCs, SMDCs also have three parts: targeting ligand, cleavable linker, and payload [310]. The main difference is that SMDCs utilize small molecule for drug localization targeting. The clinical advantages of SMDCs primarily stem from its small‐molecule characteristics, which enable superior tumor penetration without accumulating in tumors or other normal cells [311]. This reduces toxicity to normal cells while theoretically eliminating immunogenicity, thereby enhancing safety. SMDCs are gaining increasing interest as a novel alternative for drug delivery and tumor targeting applications with promising development prospects. The selection of SMDCs ligands must consider factors such as binding affinity, selectivity, and compound size. Consequently, they are primarily derived from natural ligands or their derivatives, such as folate, PSMA inhibitors, or integrin antagonists [312]. This limited selection represents the primary constraint in the current development of SMDCs. So far, most of SMDCs are still in the preclinical or clinical research stage. CBP‐1008 is a bi‐specific ligand drug conjugate targeting FRα and TRPV6 carrying MMAE as payload. The Phase I study demonstrated the manageable safety profile and promising antitumor activity of CBP‐1008 [277].

#### 7.3.2 Nb–Drug Conjugates

Nb, or VHH, is heavy‐chain‐only antibody derived from the variable region of camelids, with a molecular weight that is only about one‐tenth that of heavy‐chain antibody [313]. It is easy to express, modify, and have high stability, robust permeability, and low immunogenicity, making it an attractive choice for developing novel conjugation drugs [313, 314, 315]. NDCs are comprised of Nb as a carrier/vehicle, covalently conjugated to chemical drugs through chemical linkers. As a new generation of targeted therapeutic tools, NDCs are emerging as a key strategy to address the challenges of chemotherapy‐induced toxicity and drug resistance. Examples include VHH7‐DM1 [316], Nectin‐4‐targeting NDC [317], and PD‐L1/TLR7 dual‐targeting NDC [318].

### 7.4 Next‐Generation Functional Conjugates

#### 7.4.1 Degrader–Antibody Conjugates

DACs combine a type of degraders with a mAb via chemical linkers. Degraders include proteolysis targeting chimera, which employ diverse E3 ligases to degrade multiple protein targets and molecular glue, which can induce or stabilize the interaction between an E3 ubiquitin ligase and a target protein (substrate) and degrade the target protein [319, 320, 321]. DACs can overcome limited permeability of proteolysis targeting chimeras and minimize their on‐target off‐tumor toxicity in healthy tissues, which perform good oral bioavailability. However, poor selectivity, drug resistance, not target scaffolding proteins, and undruggable targets also have been found in DACs [12]. The first reported DAC (GNE‐987) was reported in 2020, which comprised of a mAb targeting C‐type lectin‐like molecule‐1 and a potent bromodomain‐containing protein 4 degrader [322]. After that, additional preclinical trials on DACs have been continuously conducted [323, 324]. Currently, few DACs have advanced to the clinical stage. Only Orum Therapeutics' ORM‐5029 (NCT05511844) and ORM‐6151 (NCT06419634), along with AbbVie's ABBV‐787 (NCT06068868), have initiated Phase I trials.

#### 7.4.2 Radionuclide–Drug Conjugates

RDCs consist of a targeting ligand (such as antibodies, peptides, or small molecules), a linker, a chelator, and a radionuclide [325]. Compared with conventional targeted cancer therapies, RDCs offer several unique advantages: they integrate diagnosis and therapy (theranostics) and exhibit reduced drug resistance [326]. Moreover, the linker of RDCs does not need to break upon contact with tumor cells during their action, which confers high stability and safety to RDCs in vivo while also reducing toxicity to normal tissues. At present, RDCs are gaining increasing attention worldwide, with some products already on the market and several others in the pipeline [327, 328, 329].

#### 7.4.3 Antibody–Cell Conjugates

ACCs represent a novel cellular immunotherapy first reported in 2009 [330]. They refer to the combination of immune cells with specific functions, such as natural killer cells (NK cells), cytokine‐induced killer cells (CIK cells), and mAb through linkers to together to form a coupling [331]. ACCs combine precisely targeted antibodies with cells, enabling the utilization of immune cells' innate activation signaling systems to recognize and eliminate diseased cells, thereby enhancing therapeutic efficacy. NK cells possess multiple cytotoxic mechanisms and can regulate immune responses through cytokine secretion, exhibiting effective cytotoxicity against solid tumors [332]. Researches used ACCs platform to conjugate oNK with trastuzumab and an anti‐HER2 antibody [333]. The drug called ACE1702 demonstrated cytotoxicity against HER2‐positive cancer cells in vitro and in vivo, while enhancing T cell recruitment and IFNγ secretion. CIK cells are defined as immune cells characterized by the expression of both CD3 T cell and CD56 NK cell biomarkers, which exhibit strong activity against various tumors with minimal toxicity [334]. A study showed that affixing rituximab or daratumumab to CIK cells enhances cytotoxic killing of multiple lymphoma cell lines in vitro [335]. ACE2016, which is generated by ACCs technology to conjugate donor‐derived γδ2 T cells with the EGFR‐specific antibody cetuximab, has displayed potent cytotoxicity against multiple EGFR‐positive cancer cell lines, while exhibiting minimal cytotoxic effects on normal cells [336]. The above findings collectively indicate that ACCs hold significant clinical therapeutic potential, offering a treatment approach for solid tumors that is simultaneously specific, scalable, and safe.

#### 7.4.4 Antibody–Biopolymer Conjugates

ABCs are produced by coupling mAbs with biologic polymers (such as polynucleotides, polypeptides, and polysaccharides) via molecular engineering [337]. Compared with ADCs, ABCs not only enhance tumor penetration by using the steric hindrance effect of polymer materials, but also achieve spatiotemporal‐controlled drug release in the tumor microenvironment [338]. Conjugations with polymer nano‐delivery systems lead to better solubility and immunological profile. A study found that cetuximab‐conjugated poly (γ‐glutamic acid)‐docetaxel nanomedicines exhibited efficient arrest of tumor growth [339]. Currently, research and development of ABCs for antitumor therapy are still in the preliminary stages and require further clinical trials.

### 7.5 Clinical Potential and Future Directions of Novel Conjugated Drugs

The diverse array of novel conjugated drug platforms described above holds tremendous clinical potential. ApDCs offer the advantage of low‐cost, nonimmunogenic targeting with high chemical stability. DACs provide a unique mechanism to degrade, rather than merely inhibit, disease‐causing proteins, opening up previously “undruggable” targets. SMDCs and NDCs promise superior solid tumor penetration due to their small size. RDCs uniquely integrate diagnosis and therapy, enabling patient stratification and real‐time treatment monitoring. ACCs and AOCs represent a convergence of cell therapy and gene therapy with targeted delivery, potentially creating synergistic effects that are unattainable with traditional modalities.

Looking forward, improving in vivo stability is particularly important for these novel conjugated drug platforms, especially for ApDCs, and NDCs [340, 341]. A major future direction is the development of multifunctional conjugates, such as bispecific or trispecific constructs that can engage multiple targets or simultaneously deliver a drug and a diagnostic isotope [342]. As preclinical research continues to validate these approaches, the next decade is likely to see the first approvals of DACs, SMDCs, and NDCs, significantly broadening the arsenal of targeted cancer therapies beyond ADCs and PDCs.

## 8 Conclusion, Challenges, and Future Perspectives

As a significant innovation in oncology, ADCs have achieved remarkable progress. From the inception of the “magic bullet,” through the approval of the first ADC drug Mylotarg in 2000, to the widespread application of multiple next‐generation ADCs by 2025, ADCs’ technology has undergone a lengthy journey from concept to clinical practice, now standing as a crucial pillar in cancer therapy. In recent years, ADCs’ development has not only focused on optimizing antibodies, linkers, and payloads, but has also seen the emergence of novel conjugated drugs such as bispecific ADCs, ISACs, PDCs, ApDCs, and others, significantly expanding the boundaries of targeted therapy.

The overarching objective of all conjugated drug research and development is to enhance tumor selectivity, reduce toxicity, and overcome resistance [343]. Despite these advancements, substantial clinical challenges remain.

### 8.1 Current Status and Unmet Clinical Needs for ADCs and PDCs

Despite their clinical success, both ADCs and PDCs face significant hurdles. Although ADCs can specifically target tumor cells and reduce damage to healthy tissue, the toxicity of ADCs remains a key challenge in clinical management. Major adverse events in clinical included fatigue, hematologic toxicities, nausea or vomiting, ocular toxicities, and peripheral neuropathy [344]. Off‐target toxicity, on‐target toxicity, and bystander effects on normal cells surrounding the tumor are important factors that lead to adverse events. Off‐target delivery of ADCs’ payloads and off‐target receptor‐mediated uptake of ADCs are specific mechanisms of off‐target toxicity [345]. The binding of ADCs to target antigens expressed in healthy tissues can cause on‐target toxicity. In clinical trials, dermatologic events that occur in 55% of patients treated with Padcev [346]. This adverse event is attributed to on‐target toxicity, as the ADC's target, Nectin‐4, is expressed in epidermal keratinocytes and skin appendages.

Furthermore, acquired resistance inevitably emerges, driven by mechanisms such as altered antigen expression, impaired drug transport, lysosomal dysfunction, increased drug efflux, and enhanced cancer cell survival pathways [347, 348]. For PDCs, while they offer lower immunogenicity and better tumor penetration, their primary unmet clinical need is poor circulatory stability. Peptides are rapidly degraded by proteases in the bloodstream and cleared by the kidneys, resulting in an extremely short half‐life that limits therapeutic efficacy [349]. The recent withdrawal of Pepaxto due to safety concerns in confirmatory trials also highlights the critical need for robust Phase III validation and better predictive preclinical models for PDCs.

### 8.2 Key Technological Breakthroughs Driving Next‐Generation Conjugated Drugs

To address these challenges, several key technological breakthroughs are propelling the field forward. First, developing more stable linkers (such as site‐specific conjugation techniques and tandem‐cleavage linkers) and hydrophilic payloads can optimize pharmacokinetics [350]. Second, the diversification of payloads beyond traditional microtubule inhibitors and DNA‐damaging agents is expanding therapeutic windows. Novel payloads include immunomodulators (for ISACs), radionuclides (for RDCs), protein degraders (for DACs), and oligonucleotides (for AOCs). To overcome drug resistance, novel therapeutics employing highly effective payloads with distinct mechanisms of action (such as duocarmycins and α‐amanitin) have been developed to circumvent established resistance pathways [351, 352]. Third, the use of bispecific ADCs or dual‐drug ADCs enables simultaneous targeting of multiple critical targets or signaling pathways, thereby reducing the risk of resistance arising from the loss of a single target. Fourth, advanced scaffold technologies, such as bicyclic peptides for PDCs and Nbs for NDCs, are improving target affinity and in vivo stability. Finally, CRISPR/Cas9 screens are being used to identify novel resistance genes and validate combination targets, providing a rational basis for next‐generation therapeutic strategies. Lipert et al. conducted a genome‐wide CRISPR/Cas9 screening in HER2‐positive BC patients receiving T‐DM1 therapy, successfully identifying multiple drug resistance‐associated genes with therapeutic potential [353].

### 8.3 Future Landscape: Personalized Therapies, Combination Strategies, and AI‐Driven Design

In the further future, the scope of ADCs applications will expand beyond their current use in hematological malignancies and solid tumors to encompass other disease areas, such as autoimmune diseases [354]. The future of conjugated drugs will be shaped by three major trends.

*Personalized Therapies*: The theranostic capability of RDCs exemplifies personalized medicine, where diagnostic isotopes identify patients most likely to respond before therapeutic dosing [325]. In the future, patient‐specific targeting ligands or patient‐derived xenograft models could be used to select or design optimal conjugates [355, 356].

*Combination Strategies*: Conjugated drugs will increasingly be used in rational combinations with ICIs, chemotherapy, or targeted therapies to overcome resistance and enhance antitumor immunity [357]. Recently, the use of payload binding antibodies for “inverse targeting” has been developed to improve the therapeutic selectivity of ADCs. Payload binding selectivity enhancers are coadministered with ADCs to enable the binding, neutralization, and clearance of released payload from plasma, decreasing the exposure of released payload in tissues associated with ADCs toxicities. In animal experiments, researchers observed that coadministration of 7E7‐DM4 (an anti‐CD123 DM4‐conjugated ADC) with an anti‐DM4 sdAb significantly mitigated toxic reactions (reducing weight loss from 7.9 to 3.8%) while fully preserving antitumor efficacy [358]. In another animal studies, when combining ABC3315 (a humanized Fab fragment) with polatuzumab vedotin, a significantly reduced minimum weight loss rate was similarly observed (11.86% in the ADC plus PBS treatment group vs. 4.13% in the ADC plus ABC3315 treatment group) [359].

*Artificial Intelligence (AI) in Design*: AI and machine learning are poised to revolutionize conjugate design by predicting optimal peptide sequences, linker chemistry, payload combinations, and even patient stratification [338, 360, 361]. AI can rapidly screen vast chemical spaces to predict stability, efficacy, and toxicity, thereby accelerating the development of safer and more potent conjugates. Furthermore, the application of AI to analyze real‐world clinical data will help optimize dosing regimens and identify biomarkers of response or resistance. For example, Tafazzol et al. utilized a multimodal real‐world data approach to uncover predictors of clinical response to TOP1 inhibitors and optimize ADCs strategies in colorectal cancer, demonstrating the potential of integrating real‐world evidence with AI to guide patient stratification and therapeutic decision‐making [355].

## 9 Conclusion

In summary, the field of conjugated drugs has evolved far beyond traditional ADCs, giving rise to a diverse portfolio of next‐generation platforms including PDCs, ApDCs, DACs, SMDCs, NDCs, AOCs, RDCs, ACCs, and ABCs. Each platform offers unique advantages and addresses specific limitations of its predecessors. While significant challenges related to toxicity, resistance, and in vivo stability remain, ongoing technological breakthroughs in conjugation chemistry, payload diversification, scaffold engineering, and rational combination strategies are rapidly overcoming these hurdles. Looking ahead, the integration of personalized medicine approaches, synergistic combination regimens, and AI‐driven design will unlock the full potential of these sophisticated therapeutic agents. As these innovations translate from bench to bedside, conjugated drugs will play an increasingly vital role not only in oncology but also in autoimmune and other diseases, offering hope to millions of patients worldwide.

## Author Contributions

RZ and SL performed literature selection, drafted the manuscript, and prepared the figures. ZZ, MN, JF, and JZ drafted partial sections of this manuscript, collected related references, and participated in the discussion. TL, MY, KW, and JZ designed the study and revised the manuscript. All authors contributed to the manuscript. All authors have read and approved the final manuscript.

## Funding

This work was supported by National Natural Science Foundation of China (82403929, 82503827 and 32471290), Natural Science Foundation of Zhejiang Province (LQ24H160007 and 2025C02114), and China Postdoctoral Science Foundation (2025T180593).

## Ethics Statement

The authors have nothing to report.

## Conflicts of Interest

The authors declare no conflicts of interest.

## Data Availability

The data used to support the findings of this study are included in the manuscript.
